# Prokaryotic communities of the French Polynesian sponge *Dactylospongia metachromia* display a site-specific and stable diversity during an aquaculture trial

**DOI:** 10.1007/s10482-024-01962-0

**Published:** 2024-04-11

**Authors:** Mathilde Maslin, Benoît Paix, Niels van der Windt, Rohani Ambo-Rappe, Cécile Debitus, Nabila Gaertner-Mazouni, Raimana Ho, Nicole J. de Voogd

**Affiliations:** 1 Univ Polynesie Française, Ifremer, ILM, IRD, EIO UMR 241, Tahiti, French Polynesia; 2 Naturalis Biodiversity Center, PO Box 9517, 2300 RA Leiden, the Netherlands; 3 Institute of Environmental Sciences (CML), Leiden University, PO Box 9518, 2300 RA Leiden, the Netherlands; 4 Faculty of Marine Science and Fisheries, Department of Marine Science, Hasanuddin University, Makassar, Indonesia; 5 IRD, Univ Brest, CNRS, Ifremer, LEMAR, 29280 Plouzané, France; 6 Institute of Biology (IBL), Leiden University, 2333 BE, PO Box 9505 Leiden, the Netherlands

**Keywords:** Holobiont, Marine sponges, Microbiome, Farming trials, Biogeography, French Polynesia

## Abstract

Dynamics of microbiomes through time are fundamental regarding survival and resilience of their hosts when facing environmental alterations. As for marine species with commercial applications, such as marine sponges, assessing the temporal change of prokaryotic communities allows us to better consider the adaptation of sponges to aquaculture designs. The present study aims to investigate the factors shaping the microbiome of the sponge *Dactylospongia metachromia*, in a context of aquaculture development in French Polynesia, Rangiroa, Tuamotu archipelago. A temporal approach targeting explants collected during farming trials revealed a relative high stability of the prokaryotic diversity, meanwhile a complementary biogeographical study confirmed a spatial specificity amongst samples at different longitudinal scales. Results from this additional spatial analysis confirmed that differences in prokaryotic communities might first be explained by environmental changes (mainly temperature and salinity), while no significant effect of the host phylogeny was observed. The core community of *D. metachromia* is thus characterized by a high spatiotemporal constancy, which is a good prospect for the sustainable exploitation of this species towards drug development. Indeed, a microbiome stability across locations and throughout the farming process, as evidenced by our results, should go against a negative influence of sponge translocation during in situ aquaculture.

**Supplementary Information:**

The online version contains supplementary material available at 10.1007/s10482-024-01962-0.

## Introduction

Sponges are ubiquitous filter-feeders involved in key ecological processes such as nutrient cycling, energy transfer towards higher trophic levels, substratum stability, and habitat for a wide range of associated marine organisms (Bell 2008; Pawlik and McMurray 2020). They have been farmed by humans since Greek antiquity, alternatively used as hygienic tools, household devices, medical instruments, artistic material, and biopolymers (Ehrlich and Worch 2007; Voultsiadou 2007). Over the past decades, sponges have been increasingly studied as prolific sources of nature-based pharmaceuticals (Sipkema et al. 2005a; Lowe et al. 2016; Maslin et al. 2021). For instance, sponge farming programs are an important practice to grow enough biomass to extract their natural products of interest while conserving natural stocks (Duckworth 2009; Bierwirth et al. 2022). To this end, in situ and ex situ trials have been conducted in a wide range of geographical zones, for example with *Neopetrosia* sp., *Stylissa massa* and *Callyspongia biru* in the Indo-Pacific region (de Voogd 2007; Schiefenhövel and Kunzmann 2012), *Axinella damicornis*, *Ircinia variabilis*, *Chondrosia reniformis,* and *Crambe crambe* in the Mediterranean Sea (van Treeck et al. 2003; Ternon et al. 2017), and *Discodermia dissoluta* in the Caribbean (Ruiz et al. 2013). Increased specialized metabolite yields were for instance observed in *Negombata magnifica* from the Red Sea (Hadas et al. 2005), *Dysidea avara* from the Mediterranean (De Caralt et al. 2010), as well as some New Zealand species (Duckworth and Battershill 2003a, 2003b), leading to important economical perspectives (Sipkema et al. 2005a; Osinga et al. 2010).

Sponges host a large diversity of microorganisms, which are often described as host-specific (Thomas et al. 2016). Together, they form a functional entity named the “sponge holobiont”, whereby both host and microbiome display tight interactions contributing to their overall functioning (Pita et al. 2018; Slaby et al. 2019). The sponge holobiont concept has allowed to better understand the role of sponge prokaryotic communities in the production of various biosynthetic pathways (Wilson et al. 2014; Harvey et al. 2015). As an example, sponge-associated bacteria can contribute to the production of bioactive compounds (Piel et al. 2004; Indraningrat et al. 2016), with chemical properties acting against biofoulers (Aguila-Ramírez et al. 2014), predators (e.g. fishes; Thoms et al. 2007; Slaby et al. 2019) or competitors (e.g. corals competing for space; Thoms et al. 2007). Sponge microbiomes may also contribute to the nutrient cycling and energy transfer roles of their host in their environment (de Goeij et al. 2017; Pita et al. 2018). In a global context of environmental changes such as ocean acidification, immune functions associated with the microbiome can influence the survivorship and the resilience of the sponge holobiont to future climate scenarios (Posadas et al. 2022). Considering these key roles, the dynamics of the microbiome diversity appears as an essential factor to be monitored during transplantation and farming programs (Mohamed et al. 2008a, b; Thomas et al. 2016). Such investigations provide a better holistic understanding of the parameters linked to the sponge health and thus, to the farming performance itself under a context of environmental pressures (Pita et al. 2018). To date, these dynamics have been monitored only under few aquaculture trials. Alpha-diversity increases of bacterial communities from *Mycale laximissa* and *Ircinia strobilina* (both collected from the Gulf of Mexico) were observed under closed aquaculture systems (Mohamed et al. 2008a, b). Another example is related to the transplantation of *Gelliodes obtusa* to fish farms in Philippines and revealed a stable bacterial community while facing eutrophic pressures (e.g. high concentrations of ammonium and phosphate; Baquiran and Conaco 2018).

The Indo-Pacific sponge *Dactylospongia metachromia* (Demospongiae: Dictyoceratida: Thorectidae) was part of a farming program for the extraction of several bioactive compounds in French Polynesia (Maslin 2022). This sponge species is known to produce two secondary metabolites of interest named ilimaquinone (Capon and MacLeod 1987) and 5-epi-ilimaquinone (Carte et al. 1985). Promising pharmaceutical properties were evidenced for these two compounds, including anticancer, antimicrobial, anti-HIV, antiparasitic, anti-diabetes and anti-inflammatory properties (Radeke et al. 1999; Lu et al. 2007; Kochanowska et al. 2008; Leary et al. 2009; Yasuhara-Bell and Lu 2010; Pereira et al. 2013; Daletos et al. 2014; Hitora et al. 2021). This species has a wide distribution range from the Red Sea to French Polynesia (Hooper et al. 2002; Lim et al. 2016; Cleary et al. 2020), suggesting an important adaptation potential to different environments. However, the microbial diversity associated with *D. metachormia* is still underexplored to date (Jeong and Park 2013; Cleary et al. 2020).

As the temporal dynamics of *D. metachromia* microbiome have never been investigated before, the present study aimed to monitor its diversity during the farming experiment conducted in French Polynesia (Maslin 2022). Subsequently, the objective was also to identify the major environmental factors (i.e. temperature, salinity, chlorophyll *a* and several nutrients) shaping the potentially-observed microbial changes. Considering the good survival and growth rates of the sponge host observed during trials in sites where it naturally occurs, we hypothesized a temporally stable microbiome. Additionally, a multi-scale biogeographical approach was conducted to have a broader understanding of the relative importance of these abiotic parameters, as well as the role of the host phylogeny. The core community associated with *D. metachromia* was also analyzed in order to look for essential sponge-related symbionts that could either be shaped by such variables or be broadly stable over time and space.

## Materials and methods

### Sampling and biological materials from the farming trials

**Fig. 1:**
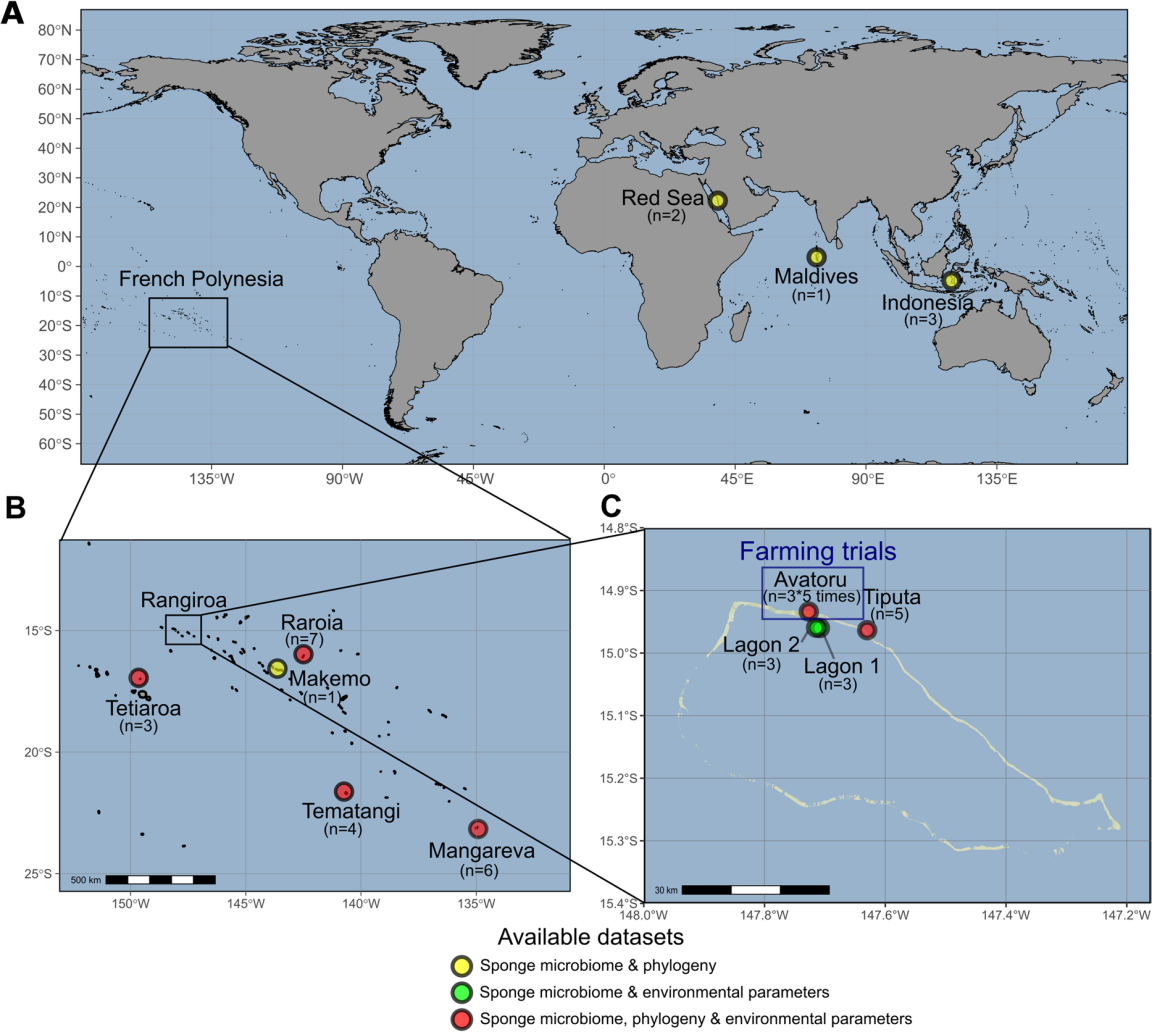
Locations of the sampling sites of the study. **A**: map of all sampling sites (Mercator projection). **B**: map of the six French Polynesian islands of the study (Tetiaroa, Rangiroa, Raroia, Makemo Tematangi and Mangareva). **C**: map of sampling sites located in Rangiroa atoll (Avatoru, Tiputa, Lagon 1 and Lagon 2). Farming trials were conducted on the Western reef side of Rangiroa, roughly 1 km away from the Avatoru channel

The farming trials were conducted at Rangiroa, an atoll from the Tuamotu archipelago. The reef site named “Avatoru” (Fig. 1) was chosen, in consultation with the maritime authorities, to perform the aquaculture trials since *D. metachromia* was found naturally growing in that area. The sponge exploitation remained under the regulations of a permanent operating license from the Direction des Ressources Marines (DRM, Tahiti) during the whole study.

The aquaculture frames were directly anchored to the reef as supports of sponge explants coming from nearby parental specimens. These structures consisted of three horizontal, table-shaped iron frames submerged between 16 and 18 m depth where the abundance of wild *D. metachromia* populations was found the highest (Fig. S1). Four different sponge explants (roughly 4*4*4 cm) were excised in December 2019 from the same original sponge. An additional piece was directly sampled as an uncultivated control, named “Donor”. The four explants were attached to the same frame using a nylon rope directly threaded through their spongin skeleton (Fig. S2). Their sampling was then carried out using SCUBA at four distinct times, spaced by 3 months. During every field survey, survival and growth rates of the new sponge generations were calculated and one explant originally coming from the same donor sponge was removed to be subsampled in triplicates. To do so, pieces of *D. metachromia* specimens were manually excised with a knife to prevent squeezing the animals and transferred into a plastic bag filled with the surrounding seawater. Only small fragments of about 2*2 cm were removed from each selected individual, leaving sufficient pinacoderm (i.e. outer layer of sponge cells) intact to allow regeneration after damage.

Samples were named according to their farming duration as follows: “9 months” for those collected in September 2020, “12 months” for December 2020, “15 months” for March 2021 and “18 months” for May 2021 (Table S1). Subsamples were cut using sterilized tweezers and scalpels for the barcoding analysis of the sponge and the metabarcoding study of the prokaryotic community. They were subsequently preserved in sterilized plastic vials filled with 96% ethanol and conserved at -20 °C until DNA extraction.

### Sampling and biological materials for the biogeographical complementary study

Different geographical scales were considered for the biogeographical study. A first approach was to conduct a global comparison, with samples collected from four different regions: the Red Sea (Saudi Arabia), the Indian Ocean (Maldives), the Makassar Strait (Indonesia, Sulawesi), and the South Pacific Ocean (French Polynesia) at different sampling times from 2014 to 2019 (Fig. 1A, Table S2). Then, a regional scale was considered by targeting three different French Polynesian archipelagos.

The sampling of French Polynesian sponges was organized during an oceanographic campaign led by the French “Institut de Recherche pour le Développement” (IRD) from 19th October to 23th November 2018 onboard the *Alis* oceanographic vessel. Subsamples were taken from donor sponge populations located at 20 m depth on the barrier reef of six different islands, encompassing more than 4000km^2^ and covering three neighboring archipelagos: Rangiroa, Makemo, Raroia, Tematangi (Tuamotu), Mangareva (Gambier) and Tetiaroa (Society) (Fig. 1B, Table S2 and Table S3).

To analyze differences at a higher geographical resolution, considering differences in terms of prevalent currents and oceanic exposition, four specific sites were chosen within the atoll of Rangiroa (Fig. 1C). Two locations named “Tiputa” and “Avatoru” were located on the external reef on the Western sides of both Tiputa and Avatoru passes. The two remaining areas named “Lagon 1” and “Lagon 2” were located within the lagoon, both facing the Avatoru pass in order to benefit from the semi-oceanic conditions allowed by the daily water exchange.

The sampling and subsampling were then processed as previously described for the study of farming trials.

### Environmental parameters

Different devices were used to record temperature and salinity conditions throughout the farming experiment in Rangiroa. First, a temperature data logger (iBee 22L Thermo Button, Plug and Track, Proges Plus, France) was directly attached to one of the frames supporting the explants, to record seasonal temperature variations every hour with an accuracy of 0,1 °C. The ThermoTrack PC V8 software was used for device calibration and data processing. Then, for salinity measurements in Practical Salinity Unit (PSU), the AquaSonde-5000 submersible multi-parameter probe (Aquaread, SDEC, France) was deployed using SCUBA at the beginning of each field survey. It was previously calibrated in the laboratory according to the manufacturer's recommendations and using the SondeLink-PC software. Lastly, for the remaining French Polynesian islands investigated in this study except Makemo due to a lack of working probes, temperature and salinity were automatically registered during each stopover using factory sensors directly operated onboard the *Alis* vessel.

For nutrient analyses, a subsample of 20 mL of seawater was fixed with 20µL of HgCl_2_ in polyethylene scintillation vials (Dominique Dutscher SAS). The vials were vigorously shaken, and then kept at 4 °C and away from light exposure. Nutrient content analyses of dissolved phosphate (PO_4_^3−^), nitrogen oxides (NO_x_), and orthosilicic acid (Si[OH]_4_) were performed by the Laboratoire des Moyens Analytiques (LAMA, Nouméa, New Caledonia) using a SEAL AA3 HR AutoAnalyzer.

For chlorophyll *a* measurement, a subsample of 500 mL of seawater was filtered on a Whatman® GF/F glass-fiber filter (0,7 nominal pore size; 47 mm diameter) to prevent the loss of picoplankton (Aminot and Rey 2001). Filters were then placed in aluminum foils and stored at -20 °C. Extraction of chlorophyll *a* from phytoplanktonic cells was performed using 90% acetone after mechanical grinding of the filters into glass tubes. The 6-mL extracts were then stored overnight in darkness at 4 °C and centrifuged the next day a few hours prior to analysis. Concentrations were measured by a Trilogy® Laboratory Fluorometer (Turner Designs) using the chlorophyll *a* Non-Acidification module. Calibration of the fluorometer was made using a Prim’Light SECOMAM® spectrophotometer.

### Barcoding of sponge samples

For phylogenetic analyses, DNA was extracted from all samples (except those from Lagon 1 and Lagon 2) using the Qiagen DNeasy Blood & Tissue kit and following manufacturer protocols for spin column extractions. The sponge 28S rRNA gene was amplified using the primers 28S-C2-fwd (5′-GAAAAGAACTTTGRARAGAGAGT-3′) and 28S-D2-rev (5′-TCCGTGTTTCAAGACGGG-3′) (Chombard et al. 1998) in a 25 µl reaction containing: 7.9 µl distilled water (sterile milliQ); 5 µl 5 × Thermo Fisher Phire Green; 1.0 µl 25 mM MgCl_2_; 1.0 µl 10 mg.ml^−1^ Life BSA; 5.0 µl 5 × Qiagen Q-solution; 1.3 µl 10 pMol.µl^−1^ forward primer; 1.3 µl 10 pMol.µl^−1^ reverse primer; 0.5 µl 2.5 mM dNTP; 0.5 µl Thermo Fisher Phire II HS Taq and 1.5 µl 0.05–0.5 ng.µl^−1^ DNA template, with the following amplification parameters: initial denature of 98℃ for 30 s, followed by 40 cycles of 98℃ for 10 s, 52.5℃ for 10 s and 72℃ for 15 s and a final extension of 72℃ for 5 min. Sponge mitochondrial cytochrome oxidase subunit 1 (COI mtDNA) gene was amplified using the primers dgLCO1490 (5′-GGTCAACAAATCATAAAGAYATYGG-3’) and dgHCO2198 (5′- TAAACTTCAGGGTGACCAAARAAYCA-3′) (Meyer et al. 2005) in a 25 µl reaction containing: 12.9 µl distilled water (sterile milliQ); 5 µl 5 × Thermo Fisher Phire Green; 1.0 µl 25 mM MgCl_2_; 1.0 µl 10 mg.ml^−1^ Life BSA; 1.3 µl 10 pMol.µl^−1^ forward primer; 1.3 µl 10 pMol.µl^−1^ reverse primer; 0.5 µl 2.5 mM dNTP; 0.5 µl Thermo Fisher Phire II HS Taq and 1.5 µl 0.05–0.5 ng.µl^−1^ DNA template, with the following amplification parameters: initial denature of 98℃ for 30 s, followed by 40 cycles of 98℃ for 10 s, 55℃ for 10 s and 72℃ for 15 s and a final extension of 72℃ for 5 min. PCR product yield was checked using Invitrogen E-gels 96 2% agarose.

PCR products were sent to Baseclear B.V. in the Netherlands for forward and reverse sanger sequencing using the same primers than those of the PCR amplification. The resulting sequences were analyzed and edited in Geneious Prime 19.2.3. Reads were assembled using a De Novo Assembly. Contigs were stripped of primer sequences and analyzed for reverse complements and quality. Sequences were aligned in Geneious using MAFFT v7.450 (Katoh et al. 2002). A phylogenetic tree was constructed using MrBayes 3.2.6 (Huelsenbeck et al. 2001). A GTR substitution model with ‘invgamma’ rate variation was preferred, setting the chain length to 1,100,000 with a subsampling frequency of 200 and a burn-in length of 100,000.

A first Mantel test was conducted to test the correlation between the phylogenetic distance matrix (based on the 28S rRNA gene) and the geographical distances matrix. The latter was obtained using the GPS coordinates of each sampling site and the *earth.dist()* function from the “fossil” R package (Vavrek 2011).

### DNA extractions, library preparation and high throughput sequencing of 16S rRNA gene amplicons

For the microbiome analysis, DNA was extracted using the FastDNA™ SPIN Kit for Soil (MP Biomedicals, Inc.) following the manufacturer instructions. Sponge tissues were cut into small pieces of approximately 3*1*0.5 mm using sterilized tweezers and scalpel blades. An extraction blank, in which no tissue was added to the tube, was also included.

The library preparation was conducted through a two-step PCR protocol for all samples, together with the extraction blank and two negative controls (mQ water instead of template DNA). For the first PCR, the V4-V5 regions of the 16S rRNA gene were targeted and amplified (Parada et al. 2016). We used 515F-Y/926R primers and the KAPA HiFi HotStart Ready Mix PCR Kit (Roche Molecular Systems, Inc.). Reactions were performed in a T100 Thermal Cycler (Bio-Rad, Hercules, CA, United States). The following thermal cycling scheme was set up: initial denaturation at 95 °C for 3 min, 30 cycles of denaturation at 98 °C for 20 s, annealing at 50 °C for 30 s, followed by extension at 72 °C for 30 s. The final extension was carried out at 72 °C for 5 min.

PCR products from the samples were checked using an E-Gel™ (agarose gels at 2%) and the absence of amplification was validated for the negative controls and the extraction blank. PCR products were then cleaned with NucleoMag NGS-Beads (bead volume at 0.9 times the total volume of the sample, Macherey Nagel, Düren, Germany) using the VP 407AM-N 96 Pin Magnetic Bead Extractor stamp (V&P Scientific, San Diego, CA, United States). Through a second PCR, 3 µL of the cleaned PCR products were then amplified and labeled using the MiSeq Nextera XTDNA library preparation kit (Illumina, San Diego, CA, United States), with the same thermal cycling scheme limited to 8 cycles. PCR products were then analyzed with the Fragment Analyzer Agilent 5300 using the DNF-910–33 dsDNA Reagent Kit (35–1,500 bp) protocol (Agilent Technologies, Santa Clara, CA, United States) to confirm successful labeling of the DNA fragments. Negative controls and extraction blanks remained negatives.

The pooling at the equimolar concentration was performed with QIAgility (Qiagen, Hilden, Germany). The final pool was then cleaned with NucleoMag NGSBeads, eluted in Milli-Q and the DNA concentration was verified using Tapestation 4150 (Kit HSD 5000, Agilent Technologies, Santa Clara, CA, United States). The sequencing was performed on an Illumina MiSeq V3 PE300 platform at BaseClear B.V. (Leiden, the Netherlands).

### 16S rRNA gene metabarcoding data processing

The raw reads were first treated by BaseClear B.V. for demultiplexing (using bcl2fastq version 2.20, Illumina) and filtering based on two quality controls (using Illumina Chastity filtering, and a PhiX control signal filtering). The following reads were then processed with the DADA2 workflow, allowing an inference to Amplicon Sequence Variant (ASV) (Callahan et al. 2016a, 2017). The “dada2” R package was used following the workflow described in Callahan et al. (2016b) and guidelines described in the online tutorial (https://benjjneb.github.io/dada2/tutorial.html). The parameters used for filtering and trimming reads were the following ones: truncation length of 290 and 240 base pairs for forward and reverse reads, respectively, maxN = 0, maxEE = 2 and truncQ = 2. The error learning step was based on 153 188 440 and 126 776 640 bases for forward and reverse reads, respectively. After the construction of the ASV table, chimeric sequences were filtered and taxonomic assignment was performed using the Silva v138 reference database (Quast et al. 2013).

The ASV and taxonomy tables produced by the pipeline were then combined into a phyloseq object, together with the sample metadata table using the “phyloseq” R package (McMurdie and Holmes 2013). The dataset was then filtered by removing all sequences affiliated to Eukaryota, chloroplast and mitochondria. Data was then decontaminated with two negative controls and the extraction blank used as control samples, using the “decontam” R package (Davis et al. 2018). The dataset was then divided into two subsets, one with the samples for the farming experiment and the second one with those for the biogeographical study. The sampling time (Donor, 9 months, 12 months, 15 months, and 18 months) and sampling sites were used as factors for the statistical analyses (Table S1 and S2).

### Metabarcoding and statistical analyses

After the filtering of reads affiliated to Eukaryota, chloroplast and mitochondria (representing together an average of 0.27% of all reads per sample, SD =  ± 0.34%), rarefaction curves performed with the “vegan” R package (Oksanen et al. 2019), reached a plateau for all samples (Fig. S3). Since the default DADA2 pipeline is performing a removal of singletons and thus considering the potential loss of very rare taxa, these rarefactions curves and the corresponding *α*-diversity metrics should be interpreted with caution. Despite the removal of these singletons, we remain confident about the relevant coverage of the richness displayed in the whole study.

For statistical analyses conducted on the biogeographical dataset, a limited number of replicates should be noticed for Maldives (n = 1), Saudi Arabia (n = 2) and Makemo (n = 2) (Table S2).

The *α*-diversity measures were estimated with Chao1 (estimated richness), Pielou (evenness) and Shannon (both richness and evenness) indices using the “ade4” and “phyloseq” R packages (Dray and Dufour 2007; McMurdie and Holmes 2013) and the rarefied datasets (rarefaction performed to the minimum library size, i.e. 31,559 and 34,103 reads, for the farming experiment and the biogeographical datasets respectively). Depending on the normal distribution assessed using a Shapiro test, the significant effect of the sampling times and sites on the diversity metrics was tested using ANOVA followed by HSD Tukey’s tests as parametric tests, as well as Kruskal–Wallis followed by pairwise Wilcoxon tests as non-parametric ones. Following recommendations for compositional approaches from McMurdie and Holmes (2014) and Gloor et al. (2017), all other analyses were conducted without rarefaction, using the phyloseq R package and the datasets normalized to the total number of sequences per sample. For both datasets, the *β*-diversity of all samples was analyzed with a non-metric multidimensional scaling (NMDS) using the Bray–Curtis dissimilarity. Differences of *β*-diversity between groups were statistically checked with one-way PERMANOVA tests followed by pairwise Adonis tests.

Distance-based Redundancy Analysis (db-RDA) using the Bray–Curtis dissimilarity was conducted to investigate the effect of environmental parameters on the *β*-diversity of the prokaryotic community of *D. metachromia*, using the “vegan” R package. A first analysis was performed with all samples from the farming dataset and tested with all available environmental parameters (temperature, salinity, PO_4_^3−^, NO_x_, Si[OH]_4_, and chlorophyll *a*). Since some parameters were not described for all sampling sites from the biogeographical study, the analysis was conducted on a restricted dataset with a selection of samples for which the temperature, salinity, PO_4_^3−^, NO_x_ and Si(OH)_4_ were measured (Table S2 and S3). This restricted biogeographical dataset includes only samples from French Polynesian sites (except Makemo as n = 1 for this site). The *ordiR2step*() function was used to verify that all these environmental factors can be selected as significant constraints to be used as explanatory variables. The overall significance of the two models was tested using the *anova.cca*() function with 999 permutations.

Following db-RDA, variance partitioning analyses (Borcard et al. 1992; Cleary et al. 2022) were conducted using the *varpart*() function from the “vegan” package for both the farming and the restricted biogeographical datasets. These analyses were performed to assess the relative percentage of the variance of the *β*-diversity explained by the selected environmental parameters, using the formula (~ temperature, ~ salinity, ~ PO_4_^3−^ + NO_x_ + Si[OH]_4_) for both datasets. Additionally, the variance of the *β*-diversity explained by environmental parameters was also assessed through a variance partitioning analysis combining the geographical distances. The geographical distances matrix was transformed using the *pcnm* function and the analysis was conducted using the following formula: (~ temperature + salinity + PO_4_^3−^ + NO_x_ + Si[OH]_4_, ~ *pcnm*[geographical.dist]$vectors).

For the biogeographical study, Mantel tests (Spearman correlation, 9999 permutations) were conducted within all samples to evaluate the correlation between the *β*-diversity distance matrix and the geographical distance matrix. Excepted for samples not barcoded (Lagon 1 and Lagon 2, Fig. 1, Table S2), an additional Mantel test was also conducted with the biogeographical dataset to assess the correlation between the *β*-diversity distance matrix and the phylogenetic distance matrix.

The "core community" was obtained using the “microbiome” R package (Lahti et al. 2017) with the *core*() function: a prevalence threshold of 90% in all samples of the “overall dataset” (including both geographical and farming datasets) and an abundance threshold of 0.01% were kept. The threshold of 90% of prevalence allowed a conservative approach to keep only persistent ASVs across space and time, but also to compare our results with previous studies using this approach (Thomas et al. 2016; Astudillo‐García et al. 2020). The terminology “core community dataset” and “overall dataset” are used below to distinguish both types of datasets. Percentages of core ASVs numbers and sequences were estimated by comparison with the total community and statistically tested using Shapiro, ANOVA and HSD Tukey’s tests.

## Results

### Environmental conditions, and sponge monitoring during farming experiment

The water temperature increased from 27.3 to 28.3 °C for farmed explants of 9 and 12 months respectively, corresponding to the beginning of the wet and warm tropical season (i.e. September to December) (Fig. S4A). The peak of 29.3 °C was reached for “15 months” samples, followed three months later by a slight decrease of -0.5 °C. Indeed, the “18 months” sampling corresponds to the start of the dry and cold season in the tropics (i.e. March–April). The salinity varied between 35.04 and 32 PSU, between December 2019 and March 2021 (i.e. “Donor” and “15 months” respectively, Fig. S4A). Yet, the average salinity dropped in May 2021 (i.e. “18 months”) to 29.9 PSU, corresponding to the return of an El Niño–Southern Oscillation climate pattern (World Meteorological Association 2022).

Regarding the nutrients, no clear trend was observed in the phosphate plot, as the averages remained very similar throughout the farming period. Nitrogen oxides and orthosilicic acid seem to vary in opposite ways (Fig. S4A), (NO_x_) concentrations being the lowest for farmed samples of “12 months” and “15 months” whereas peaks in (Si[OH]_4_) averages are noticed for the same months (i.e. December 2020 and March 2021, respectively).

Excluding the Red Sea, the Maldives, Indonesia and the atoll of Makemo (within French Polynesia) since no environmental parameters were measured for these sites (Table S2 and Table S3), spatial analyses of all environmental parameters revealed significant differences between French Polynesian islands (Kruskal–Wallis: *p* < 0.02; Table S4). Lower sea temperatures for the southern part of the Tuamotu and the Gambier archipelagos (i.e. Tematangi and Mangareva) were observed compared to warmer northern islands featuring on average 4 to 5 °C temperatures (Fig. S4B). The atoll of Rangiroa was characterized by significantly lower salinity levels (averages ranging between 31 and 33 PSU depending on the location) in contrast to the ones registered for Raroia and Tematangi, usually above 36 PSU (Pairwise Wilcoxon test, Fig. S4B). Phosphate and silicate concentrations appear to be relatively similar for all sampled sites, except for the atoll of Raroia, characterized by significantly higher concentrations compared to all sites except Mangareva and Tetiaroa (Pairwise Wilcoxon test, Fig. S4B). Finally, nitrogen oxide values fall along a north-west/south-east gradient from 0.4 µg.L^−1^ in Tetiaroa to 0.25 µg.L^−1^ in Raroia, 0.1 µg.L^−1^ in Tematangi and barely 0.05 µg.L^−1^ in Mangareva.

High and stable survival rates were observed in Avatoru throughout the farming experiment hereby addressed, ranging between 69 to 71% (Fig. S5A). For volumes, a higher peak average reaching 99 ± 59 cm^3^ after 12 months of farming can be observed, but no significant changes were determined (Fig. S5B, Kruskal–Wallis, *p* > 0.05). Our results display an annual growth rate of 100%, as the standardized initial size of each explant was set close to 50 cm^3^.

### Sponge phylogeny

Phylogenetic trees based on 28S rRNA gene and COI mtDNA sequences have been constructed using *Dactylospongia metachromia* samples collected from the farming trials and additional material from the sponge collections of Naturalis Biodiversity Center. The phylogeny of 28S rRNA genes shows some intraspecific variation with several clades forming within *D. metachromia*. Despite a significant correlation between the distance matrix based on 28S rRNA gene sequences and the geographical distances, for the whole dataset (Mantel test: r = 0.1785; *p* = 0.0241), no clear geographical pattern was noticed within the French Polynesian sites (Fig. S6). More precisely, these samples formed two different clades, with (i) Makemo, Mangareva, and Raroia samples more related to Indonesia and Red Sea samples, and (ii) samples from Rangiroa, Tematangi, and Tetiaroa. Moreover, COI mtDNA shows no intraspecific variation between all samples, with a tree forming a single clade.

### Prokaryotic diversity of farmed samples from the overall dataset

**Fig. 2:**
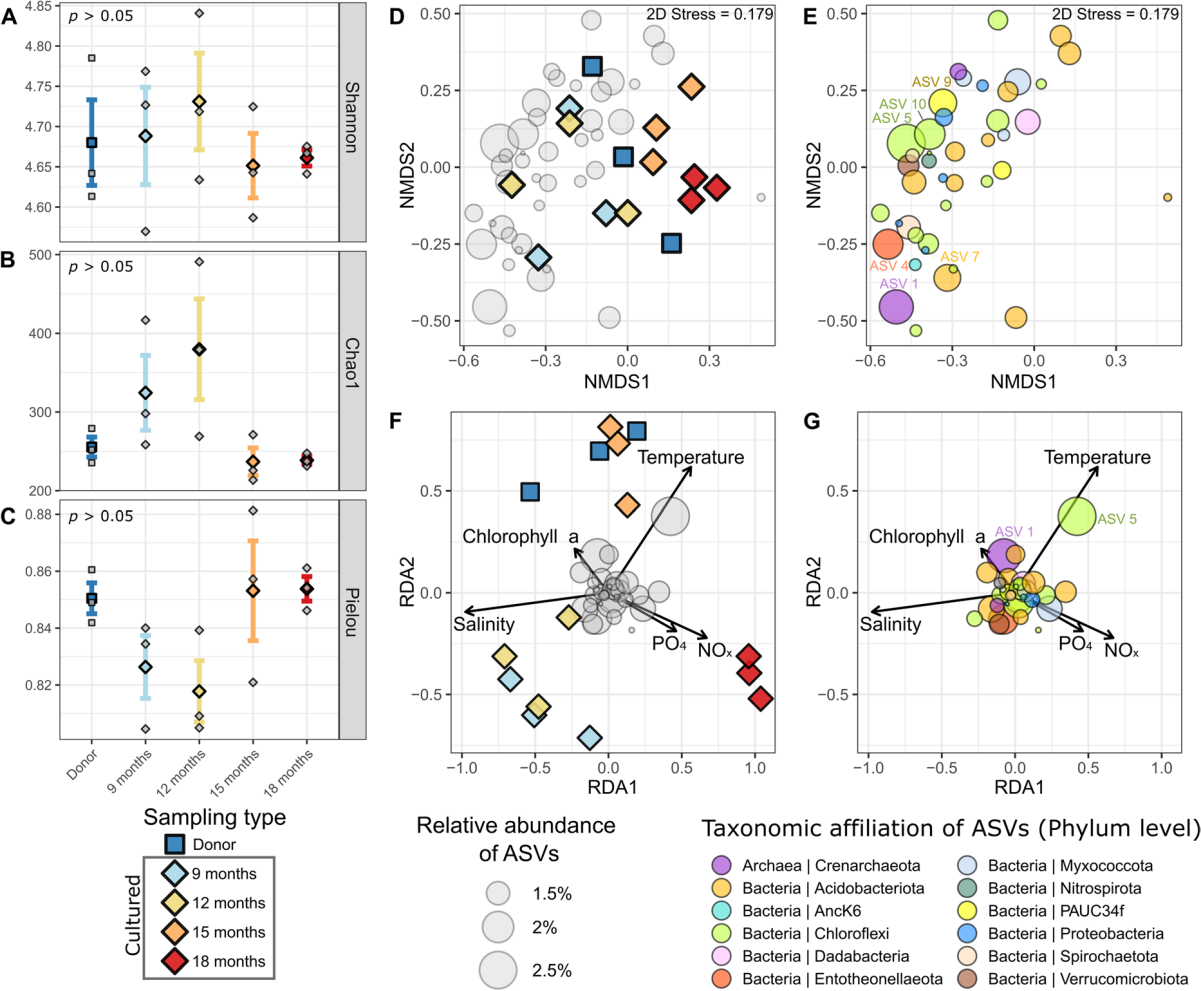
Diversity of the prokaryotic communities associated with *Dactylospongia metachromia* samples collected during the temporal study. **A**, **B** and **C**: boxplots representing the *α*-diversity metrics (**A** for Shannon, **B** for Chao1 and **C** for Pielou indices). **D** and **E**: NMDS plots representing the *β*-diversity (Bray–Curtis distances). **F** and **G**: db-RDA plots representing the environmental parameters as explanatory variables of the *β*-diversity (Bray–Curtis distances). **D** and **F**: plots of the sample scores of the NMDS and db-RDA, respectively. **E** and **G**: plots of the ASVs scores of the NMDS and db-RDA, respectively

For the farming experiment, no significant differences were observed for Shannon, Chao1, and Pielou indices (Fig. 2A, 2B and 2C, respectively) between samples collected at different time points (“donor samples”, and samples farmed during “9-”, “12-”, “15-” and “18-months”, ANOVA: *p* > 0.05 for the three indices, Table S5).

As for the NMDS analysis of *β*-diversity, distinct clusters of samples appear only for “15 months'' and “18 months” samples, while no clear patterns were observed for “Donor”, “9 months” and “12 months” samples (Fig. 2D). Despite a significant difference observed with the PERMANOVA test (*p* = 0.003), no significant pairwise differences between the sample times were noticed (pairwise Adonis: *p* > 0.05 for all comparisons). The ASVs scores of the NMDS analysis revealed major ASVs (with relative abundances > 2% on average) affiliated with several phyla including Crenarchaeota (e.g. ASV1), Chloroflexi (e.g. ASV5 and AVV10), the PAUC34f clade (e.g. ASV9), Entheonellaeota (e.g. ASV4) and Acidobacteriota (e.g. ASV7) (Fig. 2E). No clear clusters of ASVs associated with a specific phylum were observed. However, one minor ASV located on the right side of the NMDS and affiliated to the Acidobacteriota appears to be specifically associated with the cluster of “18 months” samples.

The db-RDA analysis shows three distinct clusters (Fig. 2F): (i) one gathering “Donor” and “15 months” samples, positively associated to the temperature and to a lesser extent chlorophyll *a*, (ii) one gathering “9 months” and “12 months”, positively and negatively associated to the salinity and temperature, respectively, and (iii) a last one gathering “18 months” samples, positively associated to PO_4_^3−^ and NO_x_ concentrations and negatively to the salinity (ANOVA.CCA: *p* = 0.002, Table S6B). The ASVs scores of the db-RDA analysis did not reveal ASV clusters specifically associated with environmental parameters (Fig. 2G). However, ASV5 affiliated to the Chloroflexi which happens to be amongst the dominant ones, was found positively correlated to the temperature.

The variance partitioning analysis with environmental parameters revealed that 3% of the *β*-diversity variance is explained by temperature, 10% by salinity, and 6% by nutrients (PO_4_^3−^, NO_x_ and Si[OH]_4_ together); while the percentage explained by the combination of salinity and nutrients reach 6%. Temperature, salinity and nutrients combined explain 5% of the variance (Fig. S7A).

At the family level, the prokaryotic community composition did not reveal any major changes between sample times (for the farming experiment, Fig. S7B). Most of the families observed were affiliated with three phyla: Acidobacteriota, Chloroflexi, and Proteobacteria. The main families observed in the whole dataset belong to an unidentified family from the Dehalococcoidia class (Chloroflexi phylum), the Nitrosopumilaceae family (Crenarchaeota phylum), the Caldilineaceae family (Chloroflexi phylum), an unaffiliated family from the candidate phylum PAUC34f and two unaffiliated families from the Acidobacteriae and Vicinamibacteria classes (Acidobacteria phylum). The above are respectively representing 11.5%, 6.7%, 6.0%, 6.0%, 5.5% and 4.9% of the community (SD: ± 4.5, ± 5.6, ± 2.8, ± 2.3, ± 2.3 and ± 1.8, respectively).

### Prokaryotic diversity of the biogeographical samples from the overall dataset

When focusing on the biogeographical study (Fig. S8A), significant differences between sampling sites were observed for the three *α*-diversity indices (ANOVA: *p* < 0.001, *p* = 0.001 for Shannon and Pielou, respectively; Kruskal–Wallis: *p* = 0.007 for Chao1; Table S5). For the Shannon and Chao1 indices, the highest values were observed for Tiputa samples (Fig. S8A). For the Pielou index, a more stable tendency was discerned, despite lower values observed for Raroia compared to Rangiroa (Avatoru) and Temantagi (HSD Tukey’s test, Fig. S8A).

**Fig. 3:**
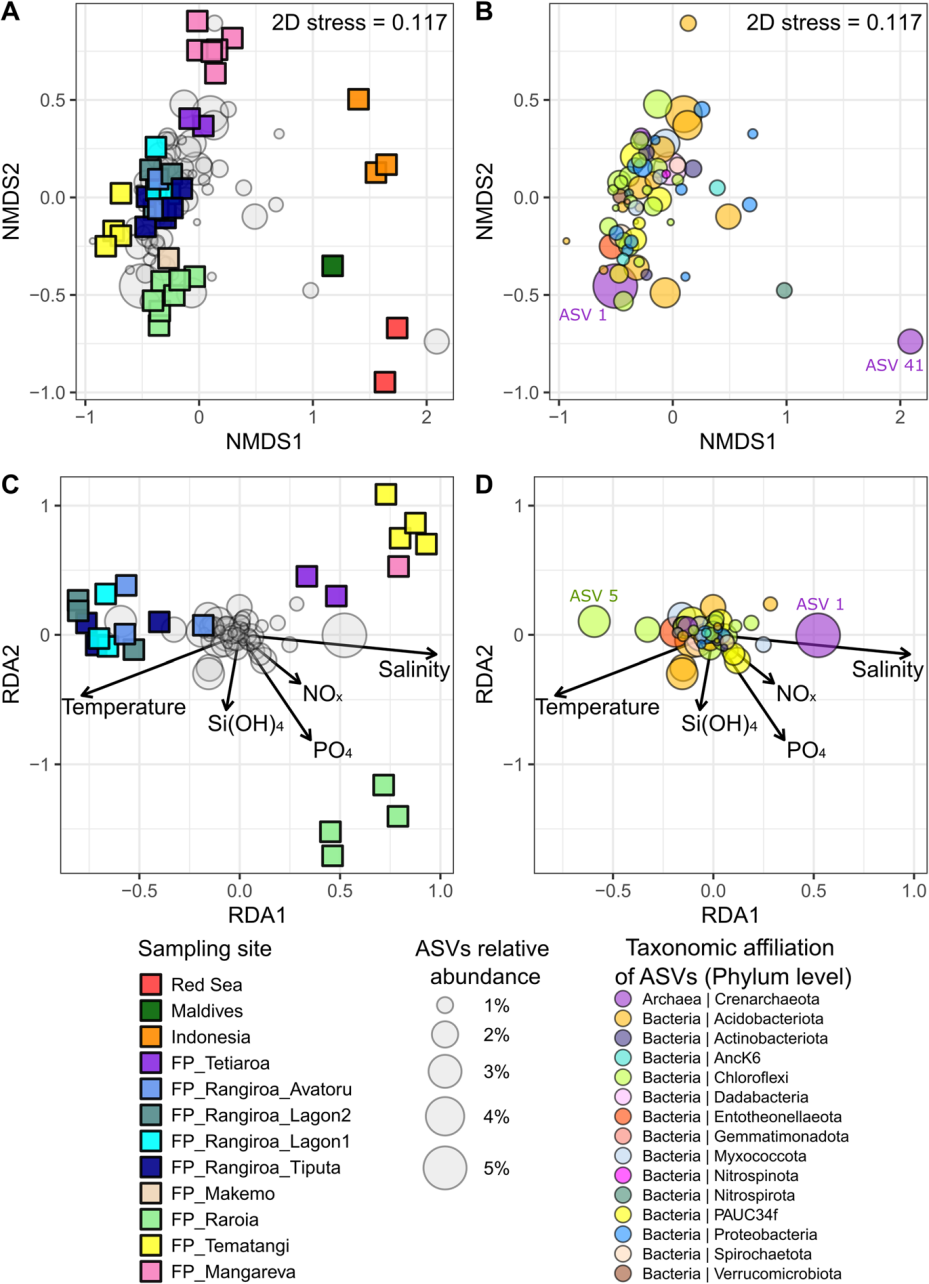
*β*-diversity of the prokaryotic communities associated with *Dactylospongia metachromia* samples from the biogeographical study. **A** and **B**: NMDS plot. **C** and **D**: db-RDA plots representing the environmental parameters as explanatory variables of the *β*-diversity. **A** and **C**: plots of the sample scores of the NMDS and db-RDA, respectively. **B** and **D**: plots of the ASVs scores of the NMDS and db-RDA, respectively

The NMDS plot of the samples from the biogeographical study (Fig. 3A) showed a distinct clustering according to sampling sites (PERMANOVA: *p* < 0.001, Table S6A), with main differences observed in the first axis (NMDS1) between French Polynesian and other sites (Red Sea, Indonesia and Maldives). Moreover, differences were observed within the French Polynesian sites on the second axis (NMDS2), with a clear clustering observed between each island (Raroia, Temantangi, Mangareva, Tetiaroa and Rangiroa). When focusing on a smaller geographical scale, with sampling sites from the Rangiroa atoll (Tiputa, Lagon1, Lagon2 and Avatoru), no significant differences of *β*-diversity were observed, except between Lagon1 and Tiputa (pairwise Adonis test). When focusing on the NMDS plot displaying the ASV scores of the biogeographical study (Fig. 3B), major ASVs were found to be notably affiliated to the Crenarchaeota (e.g. ASV1), Acidobacteriota, Chloroflexi and PAUC34f clades. While no specific patterns of ASV clusters could be associated with a specific group of samples, ASV41 affiliated with the Crenarchaeota was found to be closely related to the Red Sea samples.

Considering environmental parameters as explanatory variables (available only for French Polynesian samples, Fig. 3C and D), the db-RDA was conducted using the Bray–Curtis dissimilarity. The analysis suggested that temperature and salinity were the main parameters explaining *β*-diversity differences, together with PO_4_^3−^ concentration to a lesser extent (ANOVA.CCA: *p* < 0.001, Table S4B). Temperature and salinity seem to be positively and negatively associated with Rangiroa samples, respectively, while the opposite situation appears for Tetiaroa, Tematangi and Mangareva samples (Fig. 3C). Additionally, PO_4_^3−^ concentration was found positively associated with the *β*-diversity of Raroia samples. No specific patterns were observed within the ASV distribution on the db-RDA plot. However, two major ASVs can be noticed: (i) ASV1 affiliated to the Crenarchaeota and positively associated with the salinity, (ii) ASV5 affiliated to the Chloroflexi, mainly occurring within the Rangiroa samples and positively associated with the temperature.

A first variance partitioning analysis (Fig. S8B) revealed that temperature, salinity, and nutrients [combining PO_4_^3−^, NO_x_ and Si(OH)_4_] explain 2%, 6% and 5% of the *β*-diversity variance, respectively. More importantly, temperature and salinity combined explain 18% of the variance, while these percentages reach 3% and 8% when nutrients are combined either with the temperature or the salinity, respectively.

For the biogeographical study, a significant correlation was observed between the biogeographical and *β*-diversity (Bray–Curtis) dissimilarities (Mantel test: r = 0.8722; *p* < 0.001). Furthermore, a variance partition analysis was conducted with environmental parameters (temperature, salinity and nutrients combined) and the geographical distances as explanatory factors of the *β*-diversity variance (Fig. S8C). Results revealed that 9% of the variance is explained by the environmental parameters, 10% by the geographical distances, and 35% is explained by both datasets together. The residual variance (variance left unexplained) reached 46% for this analysis. Additionally, when comparing distance matrices from the sponge phylogeny and *β*-diversity analyses, no significant correlations were observed between the two datasets (Mantel test: r = 0.1213; *p* = 0.0693).

Finally, the prokaryotic community composition of the biogeographical samples at the family level did not reveal any major changes across space (Fig. S8D), with similar percentages of relative abundances observed for the temporal study.

### Core community of all *D. metachromia* samples

The core community dataset of *D. metachromia* was obtained by keeping only ASVs occurring at least in 90% of all samples (samples from both biogeographical and farming studies together).

**Fig. 4:**
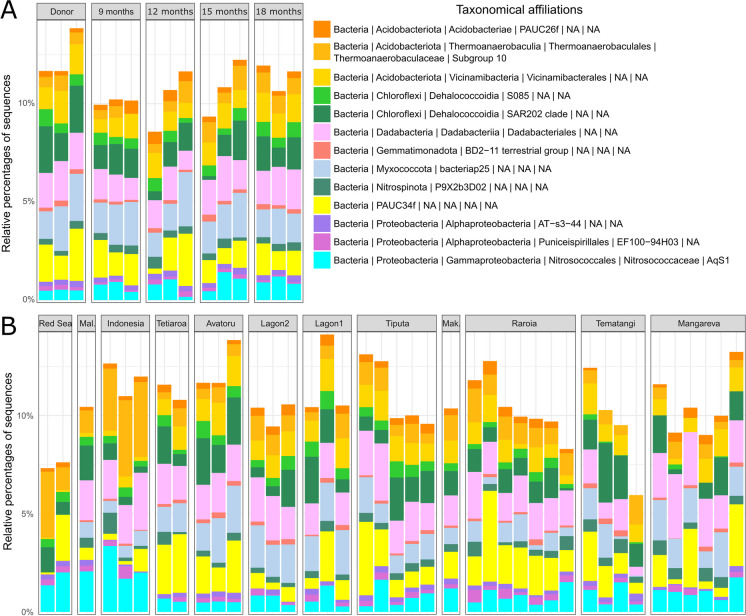
Barplots of the relative percentages of the core prokaryotic community composition (at the family level) of *Dactylospongia metachromia*. Core ASVs are present at least in 90% of the samples. **A**: samples from the farming trials (Donor: samples collected in December 2019; 9-, 12-, 15- and 18-months samples: cultured samples respectively collected in September 2020, December 2020, March 2021 and May 2021). **B:** samples from the biogeographical study (abbreviations used: Mal: Maldives, Mak: Makemo)

A total of 18 ASVs were determined as part of the core community dataset (Table S7), representing 0.3% of the total number of all ASVs in the overall dataset. The relative percentage of numbers of core ASVs per sample (Fig. S9), ranging between 4.1% and 8.8% (average 6.6%, SD ± 1.1), was not significantly different among sampling times for the farming experiment (ANOVA: *p* = 0.03, but Tukey’s test:* p* > 0.05) or among sampling sites for the biogeographical approach (ANOVA: *p* > 0.05). The relative percentage of sequences of core ASVs (Fig. 4), ranging between 5.9% and 14.1% (average 10.6%, SD: ± 2.1), did not show significant differences between sampling times (for the farming experiment, ANOVA: *p* > 0.05) or sampling sites (for the biogeographical study, ANOVA: *p* > 0.05). These sequences of the 18 core ASVs were affiliated to 13 genera (belonging mostly to unidentified families). The most abundant core ASVs were affiliated to the phyla Acidobacteriota, Chloroflexi, Dadabacteria, Myxococcota, Proteobacteria and the candidate phylum PAUC34f, representing in average 2.3%, 1.7%, 1.8%, 1.4%, 1.4%, 1.5%, of the total community, respectively (SD: ± 0.7, ± 0.8, ± 0.7, ± 0.7, ± 0.6, ± 1.0, respectively). Some of these phyla were represented by different classes such as Acidobacteriae, Thermoanaerobaculia, and Vicinamibacteria (Acidobacteria), or Alpha- and Gamma-proteobacteria (Proteobacteria).

## Discussion

As a model of interest for the production of pharmacological promising natural products (such as ilimaquinone and 5-epi-ilimaquinone), *Dactylospongia metachromia* has been the subject of an aquaculture program in French Polynesia (Maslin 2022). The production of sponge natural products and, more generally, the whole sponge physiology, is known to be intimately linked to its microbiome (Indraningrat et al. 2016; Bibi et al. 2020). When considering the launching of a sponge aquaculture industry, it thus appears essential to study the effects of transplantation and environmental changes on the whole holobiont of the targeted species (Thomas et al. 2016; Pita et al. 2018).

The microbial diversity associated with *D. metachormia* is still underexplored to date. Using a denaturing gradient gel electrophoresis fingerprinting approach, Jeong and Park (2013) described the bacterial community from Micronesian *D. metachromia* samples, while a Next Generation Sequencing method was undertaken by Cleary et al. (2020) for two *D. metachromia* samples from the Red Sea. In both studies, the most represented bacterial phyla were Actinobacteria, Proteobacteria, Chloroflexi, Acidobacteria, and Gemmatimmonadetes which are commonly abundant in so-called high microbial abundance (HMA) sponges. Based on these first observations, these sponges seem to harbor prokaryotic communities with higher diversities compared to Low Microbial Abundance (LMA) sponges (Moitinho-Silva et al. 2017).

### A temporal study revealing an overall stability of the holobiont during farming experiment, despite a moderate effect of environmental changes

The analysis of the overall prokaryotic diversity of *D. metachromia* was conducted on a temporal dimension relative to the duration of the farming experiment targeting sponge explants on a barrier reef area in Rangiroa, named Avatoru. No significant differences of *α*- diversity between the different months of farming were obtained, with a higher intra-individual variability suggesting an overall stability of the prokaryotic community. Similarly, no clear differences of *β*-diversity within the overall community were provided by the NMDS analysis. However, several environmental parameters including temperature, salinity and nutrients appear to have a slight impact within the community, notably impacting a small fraction of it [i.e. two ASVs affiliated to Crenaracheaota (ASV1) and Chloroflexi (ASV5)].

The prokaryotic community associated with marine sponges is commonly described as geographically and temporally stable. This is explained by the importance of symbiotic taxa displaying specific relationships with their sponge hosts compared to other environmental samples such as seawater, sediments or other holobionts (Hentschel et al. 2002; Taylor et al. 2004; Hill et al. 2006; Schmitt et al. 2012b; Chen et al. 2020; Hardoim et al. 2021). The HMA-LMA specificity is also linked to distinct prokaryotic composition (Campana et al. 2021b; Lesser et al. 2022), while no clear evidence of stability changes was observed over seasons and years (Erwin et al. 2015).

Studies have shown that notable differences in microbiota composition at multiple taxonomic levels exist between healthy and diseased sponges or under environmental stress (Webster et al. 2008). Sponges belonging to the order Dictyoceratida have been reported as particularly sensitive to pathogen epidemics (Smith 1941; Wulff 2001). Still, identifying the temporally stable microbial community found in the present work, no potentially harmful bacteria taxa were highlighted in the analysis of the community composition. Zaneveld et al. (2017) introduced the Anna Karenina principle for sponge-holobionts, stating that an increased dispersion of the *β*-diversity through time could be linked to multiple stress scenarios led by the emergence of diseases. In this study, the opposite situation seems to occur, with a less dispersed *β*-diversity for the last two sampling times (15 and 18 months). While the transplantation of wild sponge explants farmed in situ to closed or semi-closed systems has demonstrated important changes in the microbiota (Mohamed et al. 2008a, b), our study indicates that wild transfers can only induce moderate effects on the sponge holobiont. Such stability throughout natural contexts was also observed for other sponges, namely *Tethya bergquistae* (Cárdenas et al. 2014), *Aplysina cavernicola* (Thoms et al. 2003), *Aplysina archeri* (Hunting et al. 2013) and *Hymeniacidon heliophila* (Weigel and Erwin 2017). Additionally, a similar temporal stability was also recently described within *Hymeniacidon perlevis* and *Suberites massa* over seasonal changes (Lamb and Watts 2023).

Interestingly enough, despite highly heterogeneous ratios between ilimaquinone and 5-epi-ilimaquinone within the explants, no significant variation was either obtained in their concentrations over time or specimens during farming experiments (Maslin 2022). Together with the stable *α*- and *β*-diversity observed over time, these results corroborate the absence of a negative influence of the aquaculture system during the farming period. This finding allows us to consider a chemical exploitation in line with the positive enlargement of the sponge explants in order to seek an optimal sampling time. Nevertheless, the average concentrations of secondary metabolites of interests (ilimaquinone and 5-epi-ilimaquinone) found in the extracts of *D. metachromia* are still insufficient to match the needs of the pharmaceutical market towards new drug synthesis (Sipkema et al. 2005b; Malve 2016; Maslin 2022). Thus, the development of farming conditions allowing a temporal stability of the holobiont also needs to be complemented with an improvement of the metabolite’s extraction methodologies, to increase their yields and reach a higher potential towards medical research.

### A complementary biogeographical approach disclosing site-specific communities and confirming the importance of temperature and salinity

Values of *α*-diversity metrics were found to slightly differ between sampling sites, especially for the Red Sea samples characterized by a lower Shannon index compared to Rangiroa and Tematangi atolls. Such differences for this particular region might enlighten host-specific relationships, since for other sponges such as *Xestospongia* spp*.* the *α*-diversity did not reveal lower values for samples from Saudi Arabia compared to the Australian coast or the Indo-Pacific region (Thomas et al. 2016; Swierts et al. 2018). However, comparison of prokaryotic richness between different studies should be interpreted with caution since many methodological biases can be involved, from the molecular preparation to the data processing.

The *β*-diversity of the overall prokaryotic community associated with *D. metachromia* samples was found to be highly site-specific, whether at the global scale (when considering samples from French Polynesia together with Red Sea, Indonesia and Maldives) or within a particular region (when comparing the different French Polynesian islands). However, this specificity was found to be locally limited when assessing different sampling sites from Rangiroa. Samples from the outer part of the reef of this atoll (Tiputa and Avatoru sites) were exposed to higher currents compared to the ones located within the lagoon and only relying on daily water exchanges through the passes (sites “Lagon” 1 and “Lagon 2”). Such differences in terms of oceanographic conditions do not appear to be involved in the variations of the prokaryotic communities. A similar result was observed for the sponge *Cliona delitrix*, characterized by a prokaryotic *β*-diversity only correlated to large geographical distances (> 1000 km), whereas no significant differences were observed more locally (< 300 km) (Easson et al. 2020). However, a further look at the microbiome of *D. metachromia* with higher numbers of sites and biological replicates is needed in Indonesia, Red Sea and Maldives, to better decipher the difference on a global scale.

Many studies have provided insights on correlation between sponge phylogeny and microbiota. In some cases, dominant symbiont selection is believed to be related to sponge host identity (Easson and Thacker 2014; O’Brien et al. 2020). Several holobiont features like stress flexibility or immunity can be decisive in species adaptation, especially when considering future ocean scenarios with perturbed conditions (Posadas et al. 2022). In this sense, tropical patterns in microbial communities amongst marine sponge orders have been revealed, most probably driven not only by vertical transmission but also by abiotic factors (Taylor et al. 2007; Schmitt et al. 2012b).

Our study revealed significant correlations between geographical and *β*-diversity distances, suggesting an influence of both environmental parameters and/or phylogenetic differences between the sponge samples. However, no clear geographical pattern was observed within the phylogenetic trees and no significant correlations were observed between the host phylogeny and the *β*-diversity distances. This result suggests that the prokaryotic community could rather be shaped by the environment than by the host specificity. Similar results were observed for *Xestospongia* spp. collected from the Indo-Pacific region, the Mozambique Channel, and the Red Sea, with a prokaryotic community more influenced by the biogeography than by the host-specificity (Swierts et al. 2018). Other studies suggest a more important effect of the host-specificity (Meyer and Kuever 2008; Busch et al. 2022), especially when smaller geographical scales are considered (Cleary et al. 2018), or when the phylogenetic comparisons are covering a large sponge diversity with higher taxonomic differences (i.e. family or order levels; Yang et al. 2019). As studied for *C. delitrix*, more insight on the potential effect of host-specificity should be considered at the level of the population genetics (Easson et al. 2020). In our study, such level may justify why 10% of the *β*-diversity variance is explained exclusively by the geographical distance independently from the environmental factors. However, a large proportion of the variance associated with the environmental parameters, whether combined with the geographical distances (35%) or not (9%), confirm their predominant role driving the community structure.

When considering the relative effect of the different environmental parameters measured, the biogeographical study confirmed that temperature and salinity constitute two key abiotic factors shaping the *β*-diversity of the prokaryotic community. The effect of these parameters appears to be more important on the biogeographical study compared to the temporal one, probably in line with the greater variability of these factors through space rather than time. The temperature and salinity were found to have opposite effects on the community structure, explaining their high proportion of the shared variance. The negative relationship between temperature and salinity has been considered to play an important role within deep-sea sponge-holobionts (Busch et al. 2022), as reported for *Geodia barretti* (Strand et al. 2017; Steffen et al. 2022). In these studies, such parameters are consequently often interpreted carefully due to their collinearity with other depth-related factors, such as hydrostatic pressure or oxygen level. However, all samples in our study were collected from shallow waters with an average of 20 m (SD: ± 5 m), suggesting a limited role of the depth in the combined effect of temperature and salinity. Experimental studies have proven that an increase in temperature is one of the main factors impacting sponge holobionts health (Webster et al. 2008; Pantile and Webster 2011; Pita et al. 2018; Slaby et al. 2019). Nevertheless, it has also been observed that bacterial communities within *Ircinia* spp. can be highly stable under large fluctuations of temperature occurring in situ (Erwin et al. 2012) or in controlled conditions (Pita et al. 2013).

As for salinity changes, only few studies have looked for specific effects on sponges, especially at the holobiont scale. Although decreasing salinity gradients were linked to a decline in growth of *Cymbastela concentrica*, the abundance of its photosymbionts was not significantly affected (Roberts et al. 2006). Additionally, a stability of the host health and prokaryotic communities was demonstrated for diverse HMA and LMA species exposed to salinity disturbances over time (Glasl et al. 2018).

### Stability and role of the core community

The core concept within holobionts has been defined as the spatio-temporal stable part of the microbiome community, allowing to identify potential key symbionts displaying strong associations with their hosts (Astudillo-García et al. 2017; Risely 2020; Neu et al. 2021a). In particular, identifying the dynamics and stability of these core taxa is relevant to better understand the health and resilience of holobionts facing stress or damage, such as in a context of climate change or wild harvest for pharmaceutical valorization (Hutchins et al. 2019; Risely 2020; Neu et al. 2021a). However, the parameters to identify the core microbiome can vary a lot depending on the studies, featuring a broad set of context-based parameters, and the lack of a consensus definition of the core-community, often leading to different interpretations (Astudillo-García et al. 2017; Astudillo‐García et al. 2020). In our study, we defined the spatio-temporal core community of *D. metachromia* by identifying core ASVs with a prevalence of 90% in all samples from the overall dataset, as performed by previous studies on spatio-temporal core microbiome within sponges (Thomas et al. 2016; Astudillo‐García et al. 2020).

The threshold returned 18 core ASVs, representing a relatively small set of the overall community in terms of average percentages of sequences (10.6%) and ASV numbers (6.6%). With a threshold of 85% of occurrence, the core microbiome described within 5 sponge species was found to vary from 5 to 20 core OTUs (Thomas et al. 2016). Furthermore, a similar threshold (90% of occurrence, with an abundance threshold of 0.1%) returned 4 core OTUs for 20 host sponge species (Astudillo‐García et al. 2020). Together with these results, our work suggests that a limited number of taxa are core members of sponge microbiomes. However, the number of core ASVs or OTUs depends on sampling size, especially since larger spatio-temporal scales tend to decrease the richness of the core microbiome (Turnbaugh et al. 2007).

The core bacterial community of *D. metachromia* was found stable, with no significant changes of relative percentages of sequences and ASV numbers over time and space. Transplantation, thus, seems to have had a very limited impact on the structure of the potential key symbiotic community of the targeted sponge species. Also, the high growth rates of farmed explants could be linked to the overall stability of the core taxa, both indicating a steady fitness of the holobiont. These findings are in agreement with previous studies analyzing sponge-associated core microbiomes, either in tropical regions (Campana et al. 2021b; Leal et al. 2022), temperate waters (Lamb and Watts 2023) or polar ecosystems (Happel et al. 2022), suggesting that changing environmental conditions might have a relatively low impact in the shaping of the core sponge microbiome. The 18 core ASVs identified belong to 13 genera mostly affiliated to Acidobacteriota, Chloroflexi, PAUC34f and Proteobacteria. Such results, returning rather small globally-distributed taxa, are similar to previous works focusing on core communities defined from multiple species coming from very distinct locations (Schmitt et al. 2012a, b).

The core taxa of *D. metachromia* within the phylum Acidobacteriota, were assigned to Vicinamibacterale, Thermoanaerobaculales and the uncultured group PAUC26f. Marine Thermoanaerobaculales are not strictly sponge-related, as they have also been extracted from molluscs (Neu et al. 2021b; Banker et al. 2022). Likewise, a core ASV was affiliated to the class bacteriap25 (Myxococcales; Proteobacteria), which is found in soils and terrestrial plants such as some non-indigenous Asteraceae that have been introduced in French Polynesia (Landwehr et al. 2016; Monteiro 2020).

One of the main core ASVs belongs to the candidate phylum PAUC34f, which is described as part of the most important clades directly associated with the cycling of dissolved organic matter (DOM) within the Caribbean sponge *Plakortis angulospiculatus* (Campana et al. 2021a). Within the Nitrospinota phylum, a core ASV was affiliated to the candidate clade P9X2b3D02, which was recently also observed in *Aplysina caissara* restricted to southern Brazil (Hardoim et al. 2021). This clade did not occur in the marine sediment sampled nearby or in other coastal sponge species from the Western Atlantic. Along with some Chloroflexi and Dadabacteria taxa, Nitrospinota bacteria are known to participate in the sulfur cycling and are capable of synthesizing various B-vitamins or essential amino acids (Bayer et al. 2018; Engelberts et al. 2020). Additionally, two classes (S085 and SAR202 clades) from the phylum Chloroflexi were represented within the core ASVs. Clade SAR202 has been observed in the water column of the aphotic zone and is believed to take part in the oxidation of sulfite particles (Mehrshad et al. 2018). Recently, this clade was also found to dominate the microbiome of sponges living in extreme conditions, with large variation of pH values, temperature levels and oxygen concentrations in a semi-enclosed reef lagoon (Maggioni et al. 2023b). Such dominance of SAR202 is suggested to play an important role for providing energy to the host through their DOM assimilation ability, thus helping with its adaptation in a changing environment (Maggioni et al. 2023a, b). In respect to its potential role in DOM turnover and nutrient dynamics, especially in low-light environments, SAR202 might be providing a competitive advantage to the sponge for DOM assimilation and cycling (Bayer et al. 2018; Morganti et al. 2022).

Finally, the most abundant core ASVs belong to the order Dadabacteriales (Dadabacteria). Dadabacteria have been observed from the open ocean, from living systems related to the presence of organic carbon (including marine sponges) or from hydrothermal structures. This order notably includes obligate aerobic and photo-heterotrophic taxa; their ecological distribution throughout the water column could actually be driven by phototrophy (Graham and Tully 2021). In sponges, they have only been recently discovered (Schuster et al. 2016; Gavriilidou et al. 2023), and their ecological involvements remain partially unaddressed. Yet, they appear to degrade microbial dissolved organic matter like phospholipids and complex carbohydrates, but also to produce important compounds for the host-interaction such as vitamins and cofactors (Hug et al. 2016; Gavriilidou et al. 2023).

## Conclusion

Environmental changes often play a determinant role on the prokaryotic community of a sponge holobiont (Morrow et al. 2016; Batista et al. 2018; Busch et al. 2022). Consequently, it appears essential to better investigate the factors that could shape sponge holobiont dynamics, especially when a commercial outlet for pharmaceutics is tested during farming trials.

An overall stable *α*- and *β*-diversity of the prokaryotic community was observed for the sponge *Dactylospongia metachromia* whose explants were farmed for 18 months on the outer reef of Rangiroa (Tuamotu, French Polynesia). These results are in line with the good viability, high survival rates and constant biomass gain of the new sponge generations observed in situ*.*

However, some parameters such as temperature and salinity appear to partially impact a small fraction of the community. The moderated importance of these two abiotic factors was confirmed through a biogeographical study considering a broad regional scale (i.e. Red Sea and Indo-Pacific regions). Such a spatial investigation also revealed that both environmental variables are predominant over the effects of host-phylogeny differences. Hence, salinity and temperature need to be considered for their potential influence on the production of bioactive metabolites by the sponge-microorganism complex (Brinkmann et al. 2017).

The core microbiota of *D. metachromia* is composed of bacterial phyla widely distributed among sponges (except the poorly characterized Dadabacteria phylum) and these taxa are known for their DOM assimilation abilities, sulfur cycling and vitamin production. Such findings highlight their global importance for the holobiont functioning. Such taxa do not seem to be strongly affected either by geographical and environmental characteristics or by the duration of the farming trials. Indeed, core communities of sponge holobionts commonly appear to be highly similar alongside a broad spatio-temporal gradient.

However, thermal changes have previously been linked to bacterial shifts in sponge-associated microbiome, global issues such as a general rise in sea temperatures are likely to affect such bacterial communities through the disappearance of certain symbionts for the benefit of others (Lemoine et al. 2007; Webster et al. 2008). In the context of climate change, it becomes ever more important nowadays to keep extending our knowledge of the potential effects of ocean variables on a large diversity of sponge holobionts such as the one targeted by this work, especially considering the stability and function of their core community. In addition, our findings are of interest in a growing context of marine-based chemical substances valuation, notably leading the path to a potential exploitation of this promising sponge species in other geographical regions.

## Supplementary Information

Below is the link to the electronic supplementary material.

Supplementary file1 (DOCX 6860 KB)

## Data Availability

COI and 28S rRNA gene sequences were deposited and are publicly available in GenBank under the accession numbers OM980557-OM980598 (Table S3). 16S rRNA gene sequences were deposited and are publicly available in the NCBI Sequences Read Archive (SRA) under the BioProject ID PRJNA917856, accession number. The R script used for all the 16S rRNA gene metabarcoding analysis can be found at https://github.com/BenoitPAIX/Dactylospongia_metachromia_microbiome.
