# Supplementary material for: Prokaryotic communities of the French Polynesian sponge Dactylospongia metachromia display a site-specific and stable diversity during an aquaculture trial
Source: Antonie Van Leeuwenhoek. 2024 Apr 11;117(1):65. doi: 10.1007/s10482-024-01962-0 (PMC11008079; doi:10.1007/s10482-024-01962-0)

Supplementary information for:

**Prokaryotic communities of the French Polynesian sponge *Dactylospongia metachromia* display a site-specific and stable diversity during an aquaculture trial**

Mathilde Maslin^1,†^, Benoît Paix^2,†,^*, Niels van der Windt^2,3^,  Rohani Ambo-Rappe^4^, Cécile Debitus^5^ , Nabila Gaertner-Mazouni^1^, Raimana Ho^1^, Nicole J. de Voogd^2,3,^*

^1^ Univ. Polynesie Française, Ifremer, ILM, IRD, EIO UMR 241, Tahiti, French Polynesia

^2^ Naturalis Biodiversity Center, PO Box 9517, 2300 RA, Leiden, the Netherlands

^3^ Institute of Environmental Sciences (CML), Leiden University, PO Box 9518, 2300 RA, Leiden, the Netherlands

^4^ Faculty of Marine Science and Fisheries, Department of Marine Science, Hasanuddin University, Makassar, Indonesia

^5^ IRD, Univ Brest, CNRS, Ifremer, LEMAR, 29280, Plouzané, France

^†^ These authors contributed equally to this work as co-first authors. Both authors have the right to list their name first on their CVs.

* Corresponding authors: [benoit.paix@naturalis.nl](mailto:benoit.paix@naturalis.nl),  n.j.de.voogd@cml.leidenuniv.nl

# Supplementary tables

## Table S1. List of samples and metadata associated with the temporal study led during the farming trials performed at Avatoru (Rangiroa, French Polynesia).

| **Sample name** | **Sampling date** | **Temperature** | **Salinity** | **Chlorophyll a** | **PO_4_** | **NOx** | **Si(OH)_4_** |
| --- | --- | --- | --- | --- | --- | --- | --- |
| Donor_1 | 12.2019 | 27.7 | 35.04 | 0.155 | 0.258 | 0.139 | 1.05 |
| Donor_2 | 12.2019 | 28.9 | 32.11 | 0.43 | 0.199 | 0.182 | 0.72 |
| Donor_3 | 12.2019 | 28.9 | 32.11 | 0.43 | 0.199 | 0.182 | 0.72 |
| 09 months_1 | 09.2020 | 27.3 | 33.78 | 0.534 | 0.215 | 0.171 | 0.58 |
| 09 months_2 | 09.2020 | 27.3 | 33.78 | 0.534 | 0.215 | 0.171 | 0.58 |
| 09 months_3 | 09.2020 | 27.3 | 33.78 | 0.534 | 0.215 | 0.171 | 0.58 |
| 12 months_1 | 12.2020 | 28.3 | 34.49 | 0.169 | 0.182 | 0.07 | 1.33 |
| 12 months_2 | 12.2020 | 28.3 | 34.49 | 0.169 | 0.182 | 0.07 | 1.33 |
| 12 months_3 | 12.2020 | 28.3 | 34.49 | 0.169 | 0.182 | 0.07 | 1.33 |
| 15 months_1 | 03.2021 | 28.8 | 32 | 0.453 | 0.196 | 0.101 | 1.85 |
| 15 months_2 | 03.2021 | 28.8 | 32 | 0.453 | 0.196 | 0.101 | 1.85 |
| 15 months_3 | 03.2021 | 28.8 | 32 | 0.453 | 0.196 | 0.101 | 1.85 |
| 18 months_1 | 05.2021 | 28.6 | 29.9 | 0.198 | 0.238 | 0.222 | 0.58 |
| 18 months_2 | 05.2021 | 28.6 | 29.9 | 0.198 | 0.238 | 0.222 | 0.58 |
| 18 months_3 | 05.2021 | 28.6 | 29.9 | 0.198 | 0.238 | 0.222 | 0.58 |

## Table S2. List of the sampling sites from the biogeographical study and summary of the available metadata associated to the sites.

^a^ indicates whether the environmental parameters (temperature, salinity, PO_4_^3-^, NOx, Si[OH]^4^) were measured or not during the sampling of the sponge.

^b^ indicates whether the phylogenetic analyses were performed or not on the sponge samples.

| **Region** | **Sampling site** | **Sampling date** | **Longitude** | **Latitude** | **Depth (m)** | **Microbiome samples** | **Environmental parameters ^a^** | **28S phylogeny samples ^b^** |
| --- | --- | --- | --- | --- | --- | --- | --- | --- |
| French Polynesia | Makemo | 08.11.2018 | -143.5700 | -16.6193 | 29 | n=1 | No | Yes |
| French Polynesia | Mangareva | 28.10.2018 | -134.9352 | -23.278 | 17-22 | n=6 | Yes (n=1) | Yes (n=3) |
| French Polynesia | Rangiroa (Avatoru) | 14.09.2019 | -147.7295 | -14.9309 | 19 | n=3 | Yes | Yes (n=2) |
| French Polynesia | Rangiroa (Lagon 1) | 17.09.2019 | -147.7111 | -14.9572 | 16 | n=3 | Yes | No |
| French Polynesia | Rangiroa (Lagon 2) | 11.12.2019 | -147.7174 | -14.9566 | 22 | n=3 | Yes | No |
| French Polynesia | Rangiroa (Tiputa) | 08.12.2019 | -147.6328 | -14.9606 | 18-20 | n=5 | Yes (n=3) | Yes (n=1) |
| French Polynesia | Raroia | 05.11.2018 | -142.4236 | -16.0293 | 20-30 | n=7 | Yes (n=4) | Yes (n=3) |
| French Polynesia | Tematangi | 25.10.2018 | -140.6792 | -21.6787 | 12-26 | n=4 | Yes | Yes (n=3) |
| French Polynesia | Tetiaroa | 21.11.2018 | -149.5907 | -16.9995 | 30-40 | n=2 | Yes | Yes (n=3) |
| Indonesia | Sulawesi (Polewali) | 24.04.2018 | -4.8457 | 119.4012 | 1 | n=1 | No | Yes |
| Indonesia | Sulawesi (Karanrang) | 27.04.2018 | -4.8559 | 119.3770 | 1 | n=2 | No | Yes |
| Maldives | Faafu Atoll (Beyrufushi) | 19.02.2015 | 3.108028 | 73.0189 | 15 | n=1 | No | Yes |
| Saudi Arabia (Red sea) | Thuwal (Abu Gishaa) | 10.11.2014 | 22.255194 | 39.0256 | 21-23 | n=2 | No | Yes |

## Table S3. Detailed list of samples and metadata associated regarding the biogeographical study.

| **Sample name** | **Phylogeny analysis** | **Accession numbers (28S)** | **Temperature** | **Salinity** | **PO_4_** | **NOx** | **Si(OH)_4_** |
| --- | --- | --- | --- | --- | --- | --- | --- |
| FP_Makemo_1 | Yes | OM980567 | NA | NA | NA | NA | NA |
| FP_Mangareva_1 | Yes | OM980568 | NA | NA | NA | NA | NA |
| FP_Mangareva_2 | No | NA | NA | NA | NA | NA | NA |
| FP_Mangareva_3 | Yes | OM980569 | 23.87 | 36.011 | 0.168 | 0.074 | 0.29 |
| FP_Mangareva_4 | No | NA | NA | NA | NA | NA | NA |
| FP_Mangareva_5 | No | NA | NA | NA | NA | NA | NA |
| FP_Mangareva_6 | Yes | OM980570 | NA | NA | NA | NA | NA |
| FP_Rangiroa_Avatoru_1 | No | NA | 27.7 | 35.04 | 0.258 | 0.139 | 1.05 |
| FP_Rangiroa_Avatoru_2 | No | NA | 28.9 | 32.11 | 0.199 | 0.182 | 0.72 |
| FP_Rangiroa_Avatoru_3 | No | NA | 28.9 | 32.11 | 0.199 | 0.182 | 0.72 |
| FP_Rangiroa_Lagon1_1 | No | NA | 27.6 | 32.01 | 0.2 | 0.061 | 0.87 |
| FP_Rangiroa_Lagon1_2 | No | NA | 29.4 | 31.62 | 0.134 | 0.038 | 0.72 |
| FP_Rangiroa_Lagon1_3 | No | NA | 29.4 | 31.62 | 0.134 | 0.038 | 0.72 |
| FP_Rangiroa_Lagon2_1 | No | NA | 29.5 | 32.73 | 0.118 | 0.07 | 0.74 |
| FP_Rangiroa_Lagon2_2 | No | NA | 29.5 | 32.73 | 0.118 | 0.07 | 0.74 |
| FP_Rangiroa_Lagon2_3 | No | NA | 29.5 | 32.73 | 0.118 | 0.07 | 0.74 |
| FP_Rangiroa_Tiputa_1 | No | NA | 28.8 | 31.86 | 0.213 | 0.168 | 0.77 |
| FP_Rangiroa_Tiputa_2 | Yes | OM980597 | 28.8 | 31.86 | 0.213 | 0.168 | 0.77 |
| FP_Rangiroa_Tiputa_3 | No | NA | 28.8 | 31.86 | 0.213 | 0.168 | 0.77 |
| FP_Rangiroa_Tiputa_4 | No | NA | NA | NA | NA | NA | NA |
| FP_Rangiroa_Tiputa_5 | No | NA | NA | NA | NA | NA | NA |
| FP_Raroia_1 | Yes | OM980572 | 28.04 | 36.278 | 0.274 | 0.22 | 0.34 |
| FP_Raroia_2 | No | NA | NA | NA | NA | NA | NA |
| FP_Raroia_3 | Yes | OM980573 | 28.04 | 36.278 | 0.264 | 0.182 | 0.57 |
| FP_Raroia_4 | No | NA | NA | NA | NA | NA | NA |
| FP_Raroia_5 | No | NA | NA | NA | NA | NA | NA |
| FP_Raroia_6 | No | NA | 28.41 | 36.325 | 0.637 | 0.219 | 0.72 |
| FP_Raroia_7 | Yes | OM980574 | 28.32 | 36.325 | 0.356 | 0.275 | 5.31 |
| FP_Tematangi_1 | Yes | OM980592 | 25.14 | 36.348 | 0.165 | 0.13 | 0.21 |
| FP_Tematangi_2 | Yes | OM980593 | 25.18 | 36.275 | 0.193 | 0.102 | 0.19 |
| FP_Tematangi_3 | No | NA | 25.18 | 36.275 | 0.193 | 0.102 | 0.19 |
| FP_Tematangi_4 | Yes | OM980594 | 25.18 | 36.275 | 0.192 | 0.054 | 0.33 |
| FP_Tetiaroa_1 | Yes | OM980595 | 28.2 | 36.273 | 0.232 | 0.366 | 0.18 |
| FP_Tetiaroa_2 | Yes | OM980596 | 28.38 | 36.215 | 0.193 | 0.38 | 0.26 |
| Indonesia_1 | Yes | OM980560 | NA | NA | NA | NA | NA |
| Indonesia_2 | Yes | OM980559 | NA | NA | NA | NA | NA |
| Indonesia_3 | Yes | OM980561 | NA | NA | NA | NA | NA |
| Maldives_1 | Yes | OM980571 | NA | NA | NA | NA | NA |
| Red Sea_1 | Yes | OM980565 | NA | NA | NA | NA | NA |
| Red Sea_2 | Yes | OM980566 | NA | NA | NA | NA | NA |

## Table S4. Summary of the univariate tests results performed on the environmental parameters for the biogeographical study on French Polynesian sites and the growth of the sponges monitored during the farming trials.

**A** : results of the Shapiro tests.

**B** : results of the Kruskal-Wallis tests

| **A.** | Shapiro tests | **Variable** | **W** | **p-value** |  |
| --- | --- | --- | --- | --- | --- |
|  |  | PO_4_ | 0.68987 | 1.04E-05 |  |
|  |  | Si(OH)_4_ | 0.43251 | 2.06E-08 |  |
|  |  | NO_x_ | 0.89348 | 0.0186 |  |
|  |  | Salinity | 0.74245 | 5.25E-05 |  |
|  |  | Temperature | 0.80967 | 0.000549 |  |
|  |  | Growth | 0.88051 | 6.055e-09 |  |
|  |  |  |  |  |  |
|  |  |  |  |  |  |
| **B.** | Kruskal-Wallis tests (factor : Sampling site) | **Variable** | **chi-square** | **df** | **p-value** |
|  |  | PO_4_ | 19.016 | 7 | 0.008139 |
|  |  | Si(OH)_4_ | 15.733 | 7 | 0.02767 |
|  |  | NO_x_ | 20.504 | 7 | 0.004578 |
|  |  | Salinity | 20.718 | 7 | 0.004211 |
|  |  | Temperature | 17.308 | 7 | 0.01552 |
|  | Kruskal-Wallis test  (factor : Sampling time) | Growth | 2.3416 | 3 | 0.5046 |

## Table S5. Summary of the univariate tests results performed on the *α*-diversity metrics (Shannon, Pielou and Chao1).

Tables **A** to **C** correspond to tests performed for the biogeographical dataset.

Tables **D** to **F** correspond to tests performed for the farming trials.

|  |  |  |  |  |  |  |  |  |
| --- | --- | --- | --- | --- | --- | --- | --- | --- |
| **A.** | Shapiro tests |  | W | p-value |  |  |  |  |
|  |  | Shannon | 0.9618 | 0.1929 |  |  |  |  |
|  |  | Pielou | 0.9857 | 0.8852 |  |  |  |  |
|  |  | Chao1 | 0.94279 | 0.04298 |  |  |  |  |
|  |  |  |  |  |  |  |  |  |
| **B.** | ANOVA tests |  |  | Df | Sum Sq | Mean Sq | F value | Pr(>F) |
|  |  | Shannon | Sampling_site | 11 | 0.7762 | 0.07057 | 4.521 | 0.000601 |
|  |  |  | Residuals | 28 | 0.437 | 0.01561 |  |  |
|  |  | Pielou | Sampling_site | 11 | 0.014602 | 0.0013275 | 4.16 | 0.00111 |
|  |  |  | Residuals | 28 | 0.008934 | 0.0003191 |  |  |
|  |  |  |  |  |  |  |  |  |
| **C.** | Kruskal-  Wallis test | Chao1 |  | chi-square | Df | p-value |  |  |
|  |  |  | Sampling_site | 25.637 | 11 | 0.007347 |  |  |
|  |  |  |  |  |  |  |  |  |
| **D.** | Shapiro tests |  | W | p-value |  |  |  |  |
|  |  | Shannon | 0.95993 | 0.6912 |  |  |  |  |
|  |  | Pielou | 0.94787 | 0.4915 |  |  |  |  |
|  |  | Chao1 | 0.81997 | 0.006711 |  |  |  |  |
|  |  |  |  |  |  |  |  |  |
| **E.** | ANOVA tests |  |  | Df | Sum Sq | Mean Sq | F value | Pr(>F) |
|  |  | Shannon | Sampling_time | 4 | 0.01108 | 0.002769 | 0.424 | 0.788 |
|  |  |  | Residuals | 10 | 0.06532 | 0.006532 |  |  |
|  |  | Pielou | Sampling_time | 4 | 0.003499 | 0.0008747 | 2.501 | 0.109 |
|  |  |  | Residuals | 10 | 0.003497 | 0.0003497 |  |  |
|  |  |  |  |  |  |  |  |  |
| **F.** | Kruskal-  Wallis test | Chao1 |  | chi-square | Df | p-value |  |  |
|  |  |  | Sampling_time | 7.3333 | 4 | 0.1193 |  |  |

## Table S6. Summary of the multivariate tests results performed on the *β*-diversity analyses.

**A**: results of the PERMANOVA tests.

**B**: results of the anova.cca testing the db-RDA models.

|  |  |  |  |  |  |  |  |  |
| --- | --- | --- | --- | --- | --- | --- | --- | --- |
| **A.** |  |  | Df | Sum Of Sqs | R2 | F | Pr(>F) |  |
|  | Biogeographical dataset | Sampling site | 11 | 6.7309 | 0.69759 | 5.8717 | 0.001 |  |
|  |  | Residual | 28 | 2.918 | 0.30241 |  |  |  |
|  |  | Total | 39 | 9.6489 | 1 |  |  |  |
|  | Farming trials | Sampling time | 4 | 0.61548 | 0.42606 | 1.8558 | 0.003 |  |
|  |  | Residual | 10 | 0.82911 | 0.57394 |  |  |  |
|  |  | Total | 14 | 1.44458 | 1 |  |  |  |
|  |  |  |  |  |  |  |  |  |
| **B.** |  |  | Df | Sum Of Sqs | F | Pr(>F) |  |  |
|  | Biogeographical dataset | Model | 5 | 1.7899 | 2.5467 | 0.001 |  |  |
|  |  | Residual | 17 | 2.3897 |  |  |  |  |
|  | Farming trials | Model | 5 | 0.74065 | 1.8939 | 0.002 |  |  |
|  |  | Residual | 9 | 0.70393 |  |  |  |  |
|  |  |  |  |  |  |  |  |  |

## Table S7. Taxonomic affiliation of the 18 core ASVs determined from the biogeographical and farming datasets.

NA indicates unaffiliated taxa.

|  | **Kingdom** | **Phylum** | **Class** | **Order** | **Family** | **Genus** |
| --- | --- | --- | --- | --- | --- | --- |
| ASV_3 | Bacteria | Dadabacteria | Dadabacteriia | Dadabacteriales | NA | NA |
| ASV_13 | Bacteria | PAUC34f | NA | NA | NA | NA |
| ASV_16 | Bacteria | Acidobacteriota | Vicinamibacteria | Vicinamibacterales | NA | NA |
| ASV_20 | Bacteria | Acidobacteriota | Thermoanaerobaculia | Thermoanaerobaculales | Thermoanaerobaculaceae | Subgroup 10 |
| ASV_25 | Bacteria | Myxococcota | bacteriap25 | NA | NA | NA |
| ASV_32 | Bacteria | Chloroflexi | Dehalococcoidia | SAR202 clade | NA | NA |
| ASV_36 | Bacteria | Myxococcota | bacteriap25 | NA | NA | NA |
| ASV_63 | Bacteria | Chloroflexi | Dehalococcoidia | SAR202 clade | NA | NA |
| ASV_65 | Bacteria | Chloroflexi | Dehalococcoidia | S085 | NA | NA |
| ASV_66 | Bacteria | Proteobacteria | Gammaproteobacteria | Nitrosococcales | Nitrosococcaceae | AqS1 |
| ASV_67 | Bacteria | PAUC34f | NA | NA | NA | NA |
| ASV_76 | Bacteria | Proteobacteria | Gammaproteobacteria | Nitrosococcales | Nitrosococcaceae | AqS1 |
| ASV_80 | Bacteria | Nitrospinota | P9X2b3D02 | NA | NA | NA |
| ASV_82 | Bacteria | Acidobacteriota | Acidobacteriae | PAUC26f | NA | NA |
| ASV_121 | Bacteria | Proteobacteria | Alphaproteobacteria | AT-s3-44 | NA | NA |
| ASV_123 | Bacteria | Proteobacteria | Alphaproteobacteria | Puniceispirillales | EF100-94H03 | NA |
| ASV_160 | Bacteria | Gemmatimonadota | BD2-11 terrestrial group | NA | NA | NA |
| ASV_214 | Bacteria | Chloroflexi | Dehalococcoidia | SAR202 clade | NA | NA |

# Supplementary figures

## Figure S1. Photographs of *Dactylospongia metachromia* specimens collected for the farming trials.

**A** and **B**: excised “Donor” individual for the farming trial.

**C**: farming table.

**D**, **E**, **F** and **G**: growing explants collected after 9, 12, 15 and 18 months, respectively


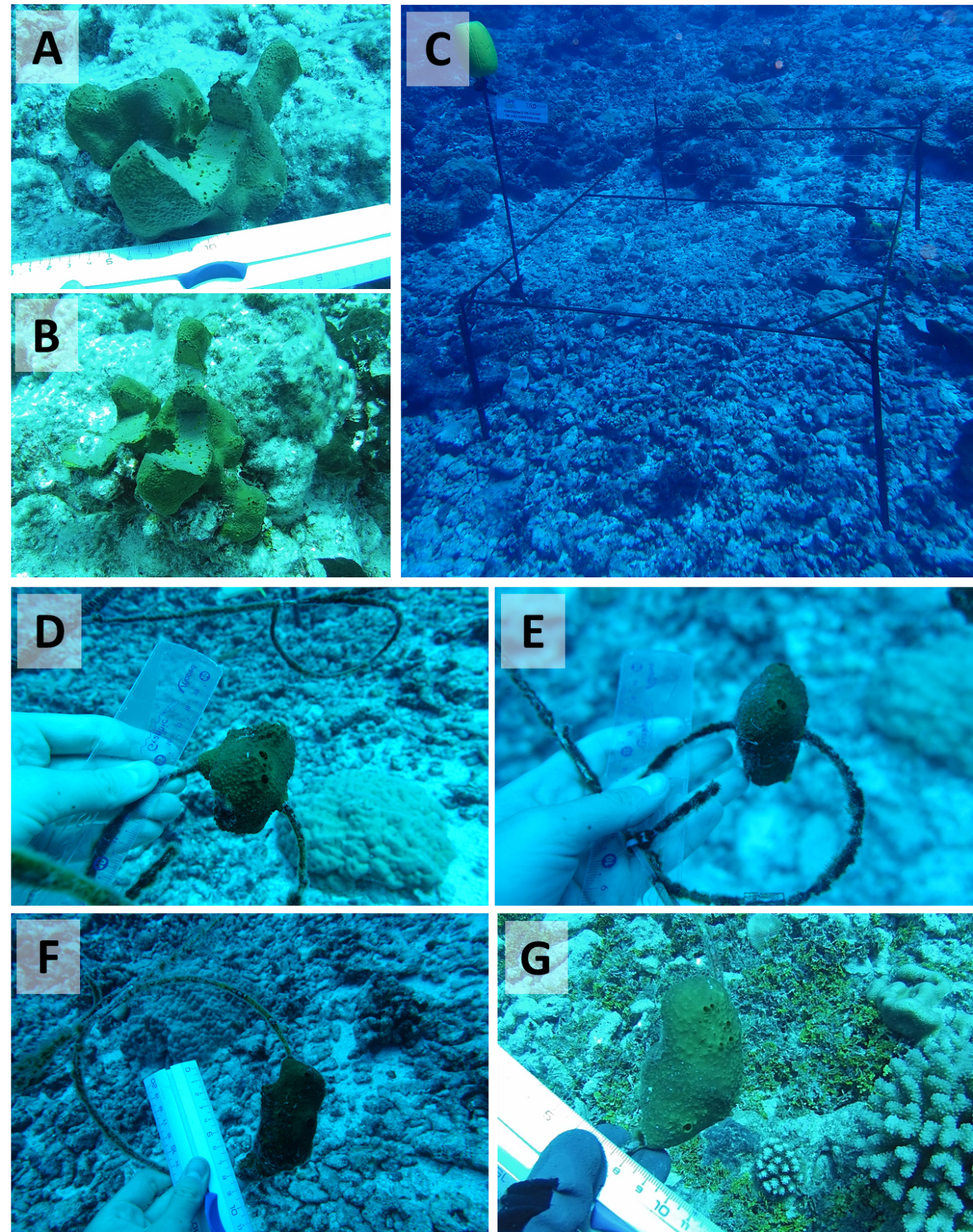


## Figure S2. Schema of the horizontal frames used for the farming trials.

**
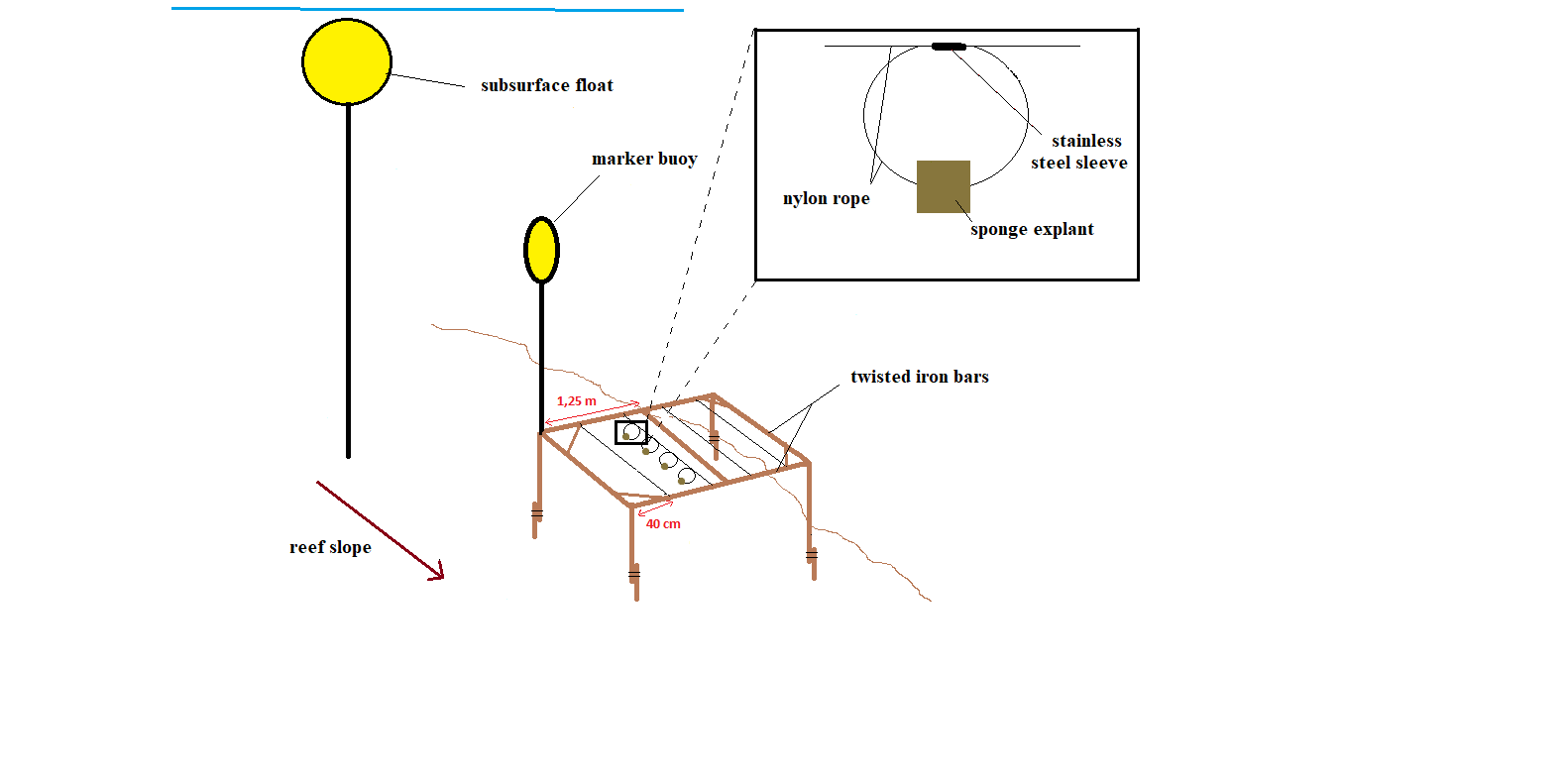
**

## Figure S3. Rarefaction curves obtained after the data processing of the 16S rRNA gene sequences with the DADA2 pipeline and the filtration of sequences affiliated to eukaryotes, chloroplasts and mitochondria.


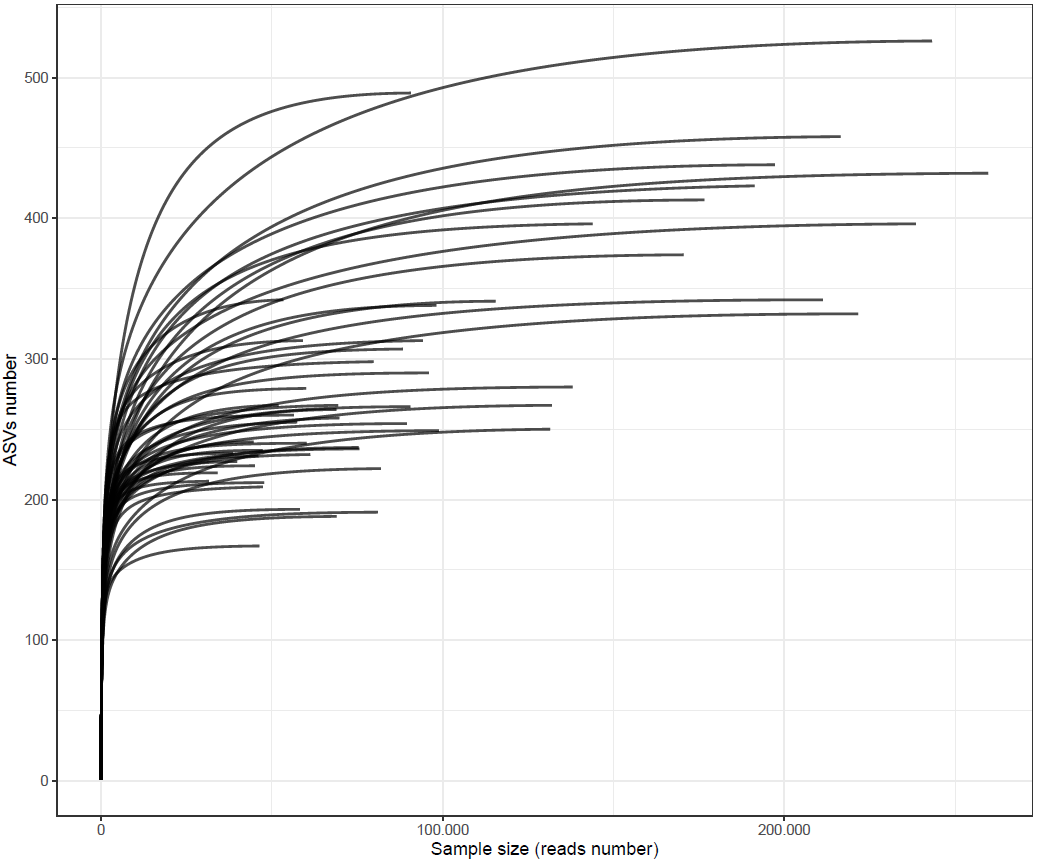


## Figure S4. Plots of the average and standard errors of the environmental parameters measured.

**A**: samples from the farming trials.

**B**: samples from the biogeographical study. Units : Temperature in °C, Salinity in PSU, [PO_4_^3-^], [NO_x_] and [Si(OH)_4_] in µM and [Chlorophyll *a*] in µg.L^-1^. Lowercase letters from a to d indicate the results from the pairwise Wilcoxon tests.


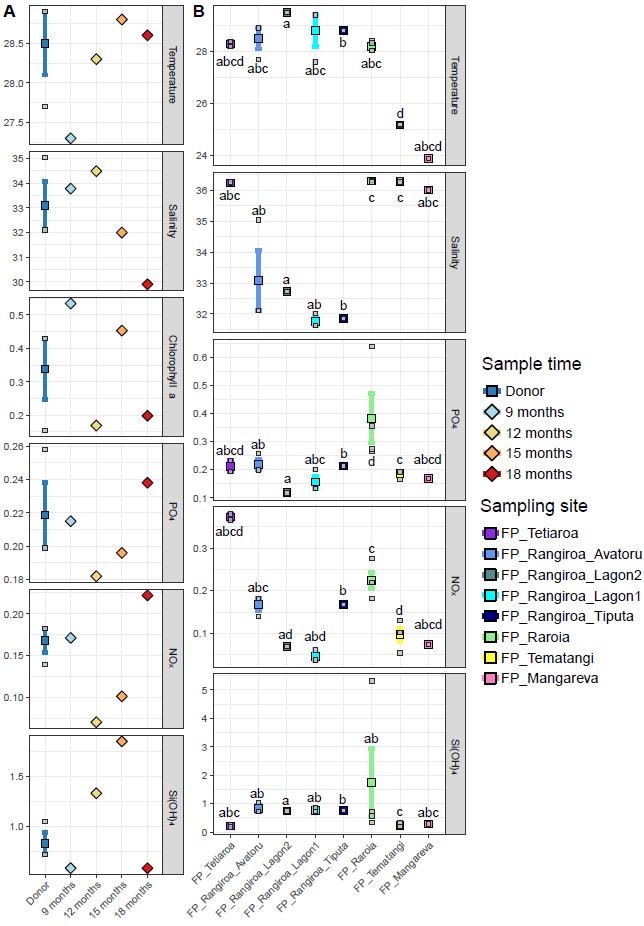


## Figure S5. Survival rates (A) and volumes (B) of *Dactylospongia metachromia* explants during the farming study.


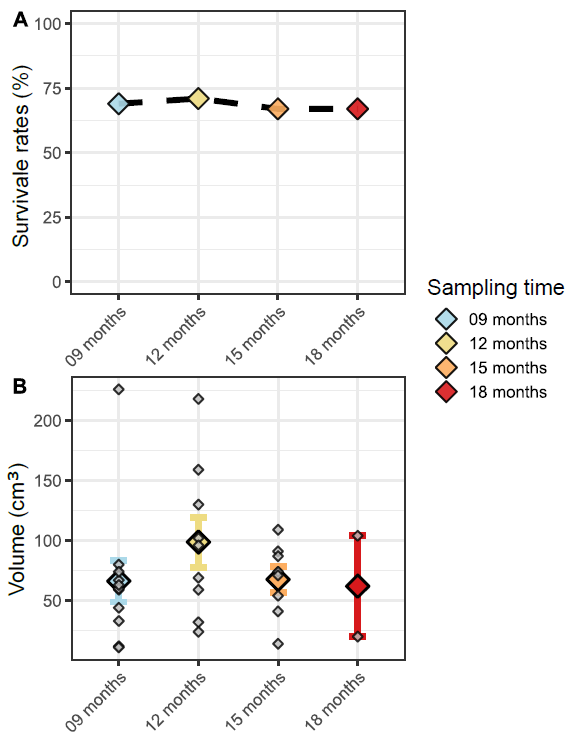


## Figure S6. Phylogenetic tree resulting from the Bayesian analysis of 28S rRNA gene sequences from all *Dactylospongia metachromia* samples used for this study and a *Dactylospongia* sp. outgroup.

Node support values indicate posterior probability values. For the phylogenetic analysis, the tree was enriched with additional 28S rRNA gene sequences from *Dactylospongia* samples of the Naturalis Biodiversity Center collection, named “RMNH.POR”.


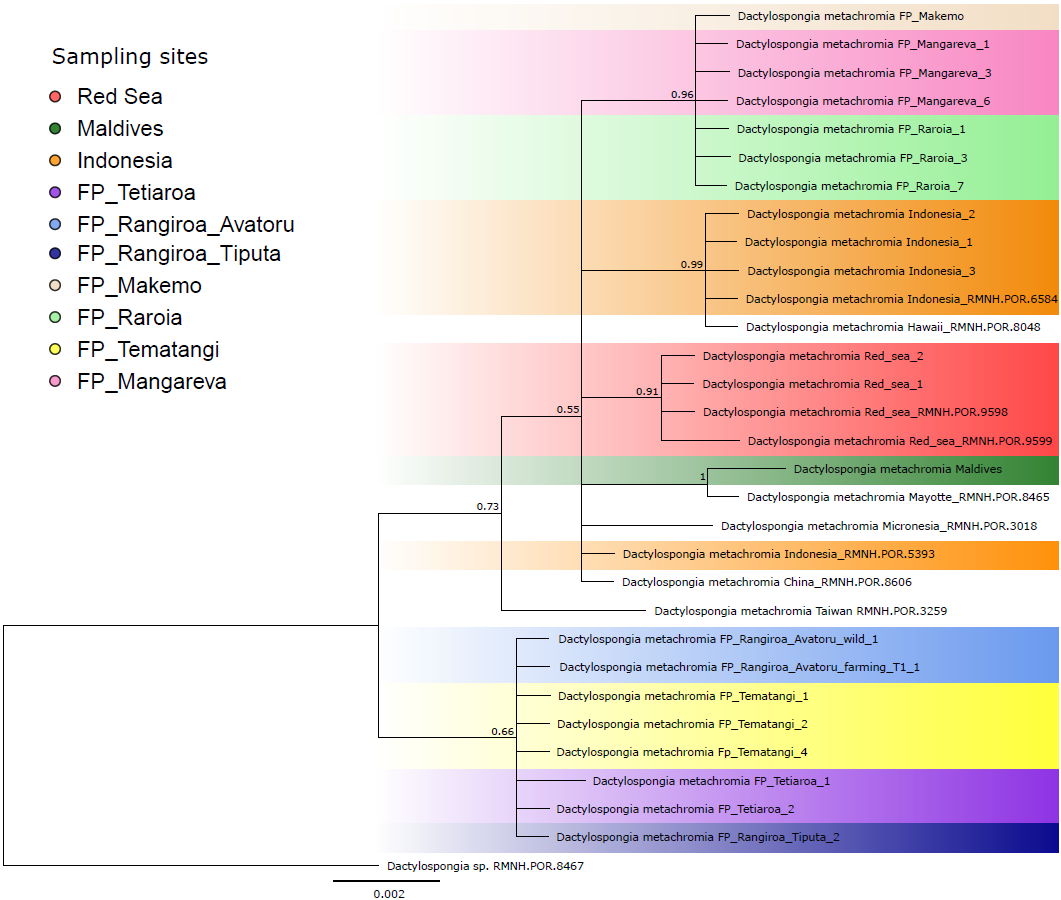


## Figure S7. Supplementary analyses of the prokaryotic diversity of *Dactylospongia metachromia* samples from the temporal study (farming trials).

**A**: variance partitioning analysis for the farming experiments, comparing the percentages explained by temperature, salinity and nutrients (combining PO_4_, NOx and Si[OH]_4_).

**B**: barplots of the relative percentages of the total prokaryotic community composition (at the family level) of *Dactylospongia metachromia*. “Other” refers to families below 3%.


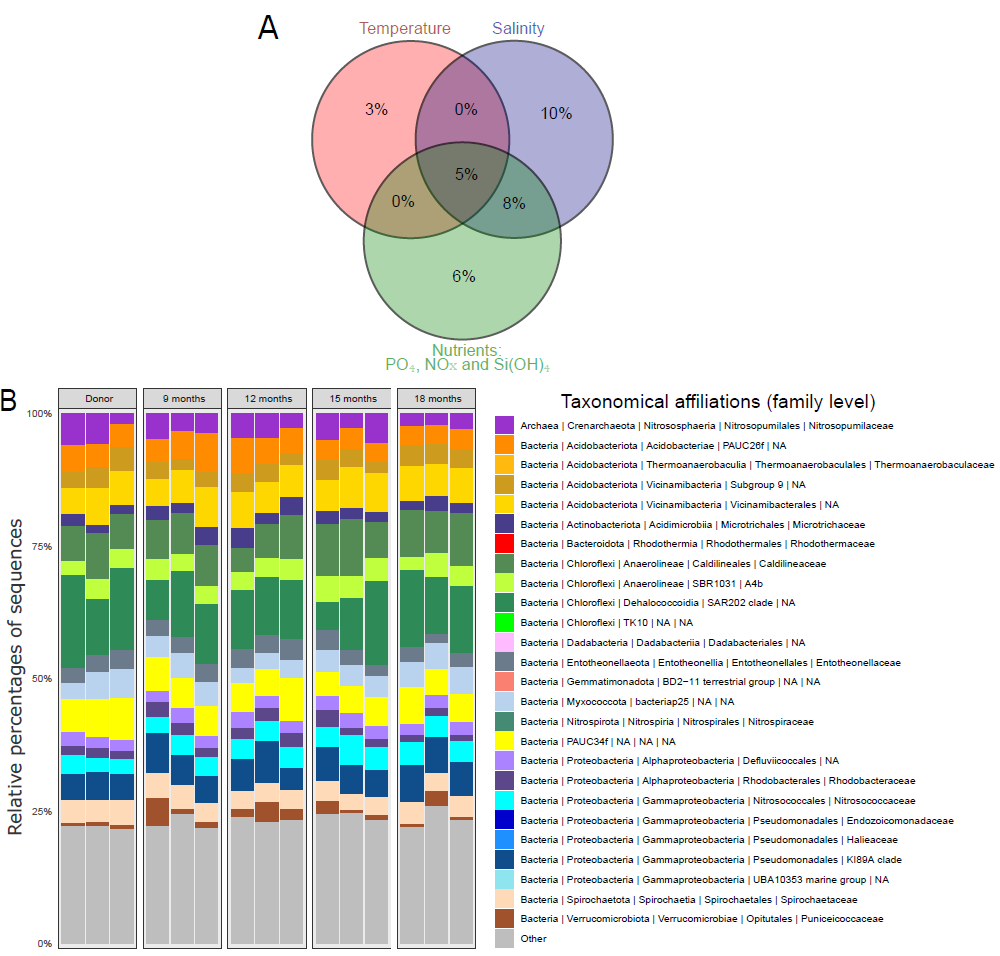


## Figure S8. Supplementary analyses of the prokaryotic diversity of *Dactylospongia metachromia* samples from the biogeographical study.

**A:** plots of the average and standard errors of α-diversity metrics (Shannon, Chao1 and Pielou) for prokaryotic communities associated with *Dactylospongia metachromia*. *p* values and lowercase letters from a to d indicate the results from the ANOVA and HSD Tukey’s tests for Shannon and Pielou indexes, together with Kruskal-Wallis and pairwise Wilcoxon tests for Chao1.

**B:** variance partitioning analysis for the biogeographical study, comparing the percentages explained by temperature, salinity and nutrients [combining PO_4_, NO_x_ and Si(OH)_4_].

**C:** variance partitioning analysis for the biogeographical study, comparing the percentages explained by environmental parameters [combining temperature, salinity, PO_4_, NO_x_ and Si(OH)_4_] and geographical distances between sampling sites.

**D:** barplots of the relative percentages of the total prokaryotic community composition (at the family level) of *Dactylospongia metachromia*. “Other” correspond to families below 3% (Abbreviations: Mal. - Maldives, Mak. - Makemo).


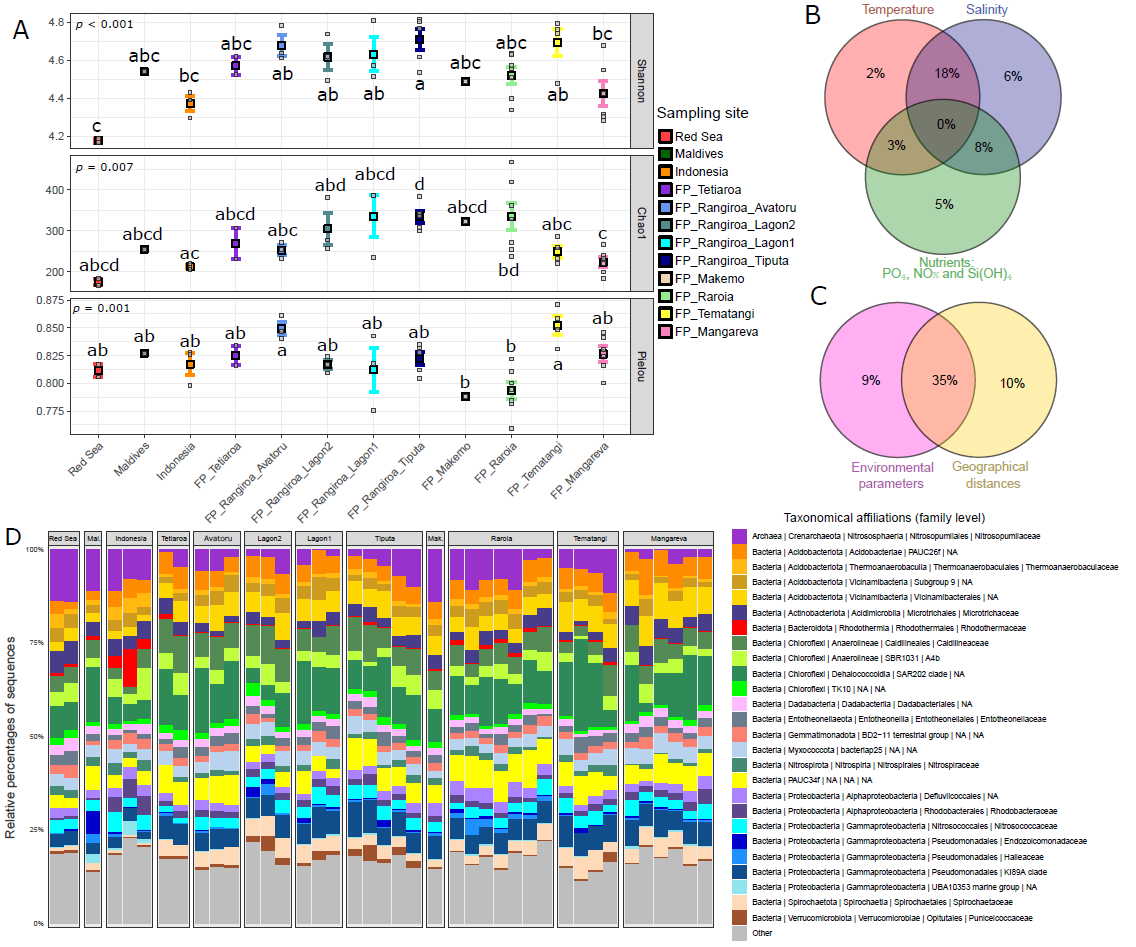


## Figure S9. Boxplots representing the proportion of the observed richness of the core community (number of core ASVs) of *Dactylospongia metachromia* compared to the total observed richness (total number of ASVs). A

**A**: samples from the farming trials.

**B**: samples from the biogeographical study.


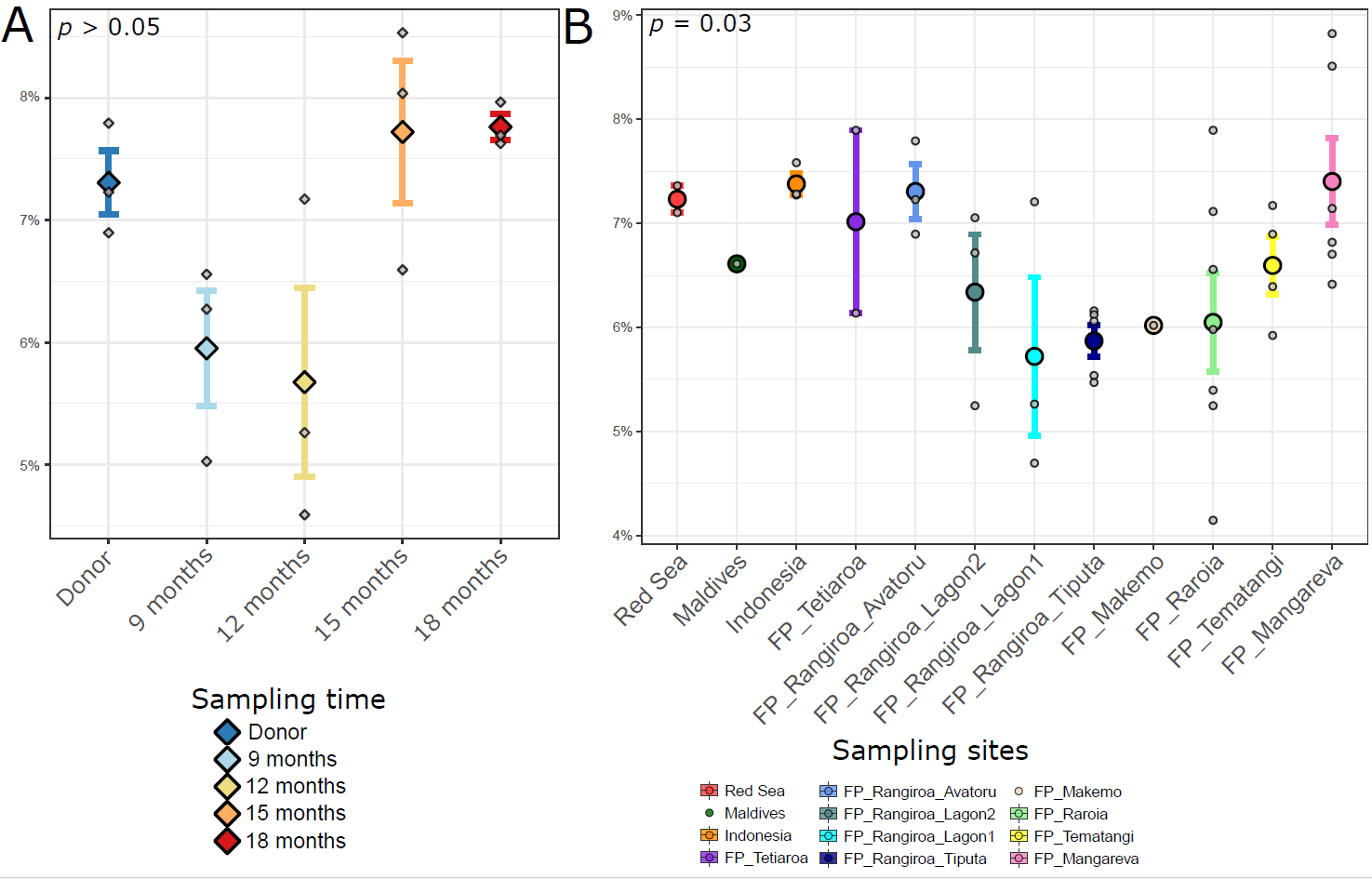

Supplement: Supplementary file 1 — Supplementary file1 (DOCX 6860 KB) [file 10482_2024_1962_MOESM1_ESM.docx]
